# Drugs repurposed for COVID-19 by virtual screening of 6,218 drugs and cell-based assay

**DOI:** 10.1073/pnas.2024302118

**Published:** 2021-07-07

**Authors:** Woo Dae Jang, Sangeun Jeon, Seungtaek Kim, Sang Yup Lee

**Affiliations:** ^a^Metabolic and Biomolecular Engineering National Research Laboratory, Department of Chemical and Biomolecular Engineering (BK21 four), KAIST Institute for the BioCentury, Korea Advanced Institute of Science and Technology (KAIST), Daejeon 34141, Republic of Korea;; ^b^Systems Metabolic Engineering and Systems Healthcare Cross-Generation Collaborative Laboratory, KAIST, Daejeon 34141, Republic of Korea;; ^c^Zoonotic Virus Laboratory, Institut Pasteur Korea, Seongnam 13488, Republic of Korea;; ^d^KAIST Institute for Artificial Intelligence, BioProcess Engineering Research Center, BioInformatics Research Center, KAIST, Daejeon 34141, Republic of Korea

**Keywords:** cell-based assay, drug combinations, drug repurposing, SARS-CoV-2, docking-based virtual screening

## Abstract

The COVID-19 pandemic caused by SARS-CoV-2 is an unprecedentedly significant health threat, prompting the need for rapidly developing antiviral drugs for the treatment. Drug repurposing is currently one of the most tangible options for rapidly developing drugs for emerging and reemerging viruses. In general, drug repurposing starts with virtual screening of approved drugs employing various computational methods. However, the actual hit rate of virtual screening is very low, and most of the predicted compounds are false positives. Here, we developed a strategy for virtual screening with much reduced false positives through incorporating predocking filtering based on shape similarity and postdocking filtering based on interaction similarity. We applied this advanced virtual screening approach to repurpose 6,218 approved and clinical trial drugs for COVID-19. All 6,218 compounds were screened against main protease and RNA-dependent RNA polymerase of SARS-CoV-2, resulting in 15 and 23 potential repurposed drugs, respectively. Among them, seven compounds can inhibit SARS-CoV-2 replication in Vero cells. Three of these drugs, emodin, omipalisib, and tipifarnib, show anti-SARS-CoV-2 activities in human lung cells, Calu-3. Notably, the activity of omipalisib is 200-fold higher than that of remdesivir in Calu-3. Furthermore, three drug combinations, omipalisib/remdesivir, tipifarnib/omipalisib, and tipifarnib/remdesivir, show strong synergistic effects in inhibiting SARS-CoV-2. Such drug combination therapy improves antiviral efficacy in SARS-CoV-2 infection and reduces the risk of each drug’s toxicity. The drug repurposing strategy reported here will be useful for rapidly developing drugs for treating COVID-19 and other viruses.

## Significance

Recent spread of SARS-CoV-2 has sparked significant health concerns of emerging infectious viruses. Drug repurposing is a tangible strategy for developing antiviral agents within a short period. In general, drug repurposing starts with virtual screening of approved drugs employing docking simulations. However, the actual hit rate is low, and most of the predicted compounds are false positives. To tackle the challenges, we report advanced virtual screening with pre- and postdocking pharmacophore filtering of 6,218 drugs for COVID-19. Notably, 7 out of 38 compounds showed efficacies in inhibiting SARS-CoV-2 in Vero cells. Three of these were also found to inhibit SARS-CoV-2 in human Calu-3 cells. Furthermore, three drug combinations showed strong synergistic effects in SARS-CoV-2 inhibition at their clinically achievable concentrations.

Severe acute respiratory syndrome coronavirus 2 (SARS-CoV-2) is a novel coronavirus that causes coronavirus disease 2019 (COVID-19) (1). The World Health Organization (WHO) first declared COVID-19 a global health emergency in January 2020. The virus has spread to almost all countries worldwide with cases identified in Asia, Europe, North and South America, Australia, and Africa. The total number of confirmed global COVID-19 cases as of May 23, 2021 is 166,723,247 with 3,454,602 deaths (https://coronavirus.jhu.edu/map.html). Institutions and companies around the world have been exerting much effort in rapidly developing vaccines and drugs to fight COVID-19. Recently, several vaccine candidates showed promising results. In addition to vaccines, it is necessary to rapidly develop therapeutic drugs as we are now observing the second wave of COVID-19 (2). More importantly, we need to establish a general strategy for rapidly developing drugs for treating other infectious viruses that might emerge in the future.

The SARS-CoV-2 virus is a member of the Coronaviridae family of viruses containing a single-stranded positive-sense RNA genome encapsulated within a membrane envelope. SARS-CoV-2 belongs to the genus *Betacoronavirus*, which also includes severe acute respiratory syndrome coronavirus (SARS-CoV) and Middle East respiratory syndrome coronavirus (MERS-CoV). SARS-CoV-2 contains at least four structural proteins: Spike (S) protein, envelope (E) protein, membrane (M) protein, and nucleocapsid (N) protein. Among them, the S protein, a surface-located trimeric glycoprotein of coronaviruses, promotes attachment of viruses to the host cells through binding to angiotensin converting enzyme 2 (ACE2) and virus-cell membrane fusion during viral infection (3). Thus, the S protein has been considered as a major target for the development of vaccines and therapeutics against SARS-CoV-2 (4).

**Fig. 1.**
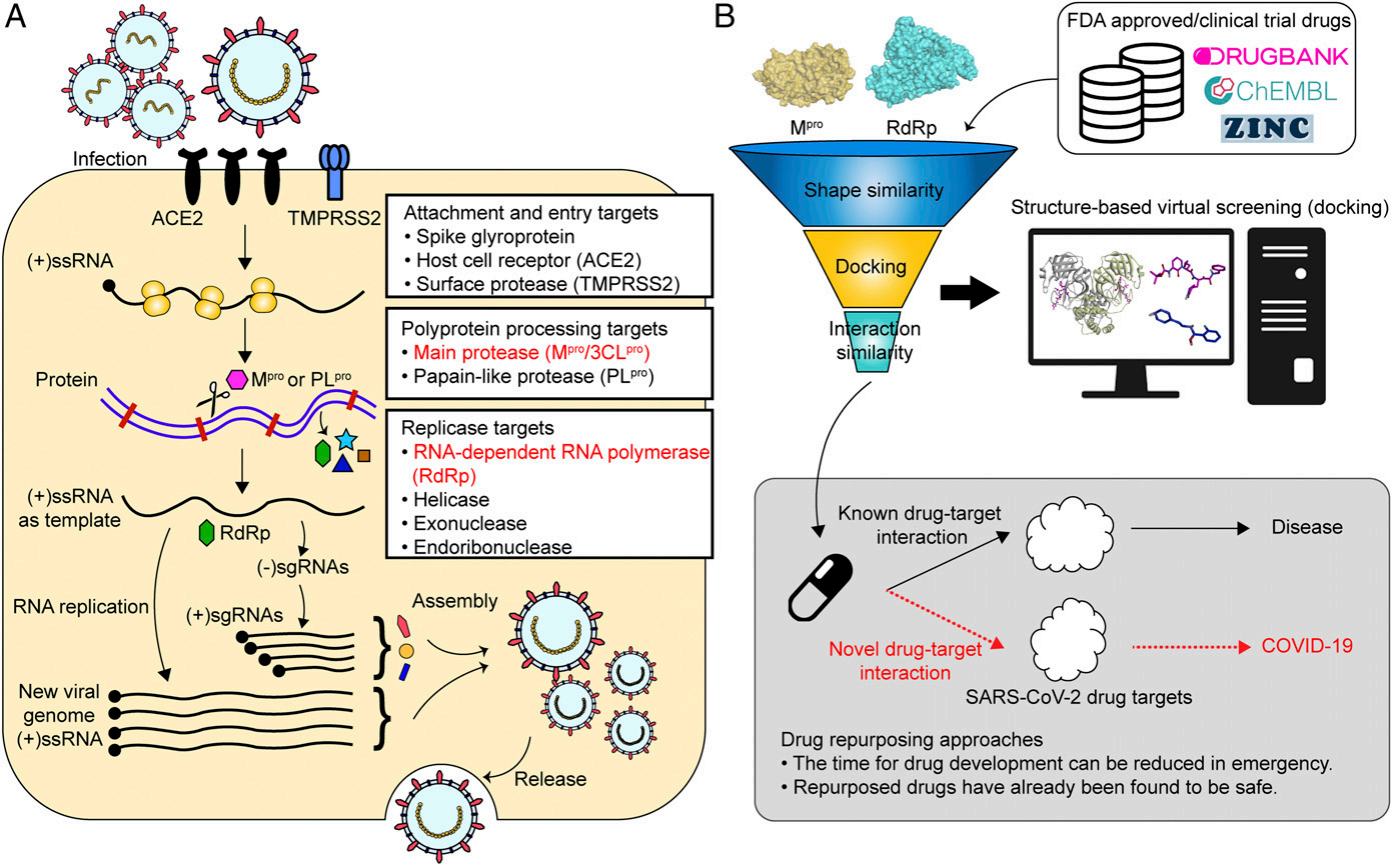
Drug targets against SARS-CoV-2 and computational drug repurposing strategy. (*A*) Potential drug targets in SARS-CoV-2 replication cycle. Targets for viral attachment and entry include the viral spike glycoproteins, host receptors (ACE2), and proteases (TMPRSS2). Polyprotein processing can be targeted by inhibiting viral proteases such as main protease M^pro^ and papain-like proteases. Viral replicase-related enzymes are also attractive drug targets for antiviral activity. RdRp and helicase are important enzymes involved in the transcription and replication of SARS-CoV-2. Among these, the most important and less variable M^pro^ and RdRp were selected as drug targets in this study. (*B*) Docking-based virtual screening can identify novel compounds against targets of SARS-CoV-2 among the collection of approved and clinical trial drugs. Computational drug repurposing is an effective approach to identify novel drug-target interactions using the drugs already known to be safe, which provides the advantages of significantly reducing time for drug development and reduced failure rate.

Previous research effort to develop antiviral agents against the members of coronavirus family suggested the ACE2 entry receptor, the RNA-dependent RNA polymerase (RdRp), and the main protease (M^pro^) as suitable drug targets (Fig. 1*A*) (5–7). As there is a high chance that coronaviruses will undergo mutations to become a new infectious virus, identification of promising targets for antiviral therapies against SARS-CoV-2 should exploit the structural similarities among different coronaviruses and focus on those proteins that are highly conserved across multiple coronaviruses. Among the several potential targets of coronaviruses, replication-related enzymes, such as RdRp and protease, are highly conserved (8, 9). Drugs that inhibit conserved proteases, such as M^pro^ and papain-like protease (PL^pro^), are capable of preventing replication and proliferation of the virus by interfering with the posttranslational processing of essential viral polypeptides (10, 11) and can also reduce the risk of mutation-mediated drug resistance. A combination of lopinavir and ritonavir targeting proteases is effective in treating HIV and SARS-CoV infections and is one of the promising drug candidates for COVID-19 treatment. However, recent clinical trials of the combination of lopinavir and ritonavir on 199 patients showed no significant therapeutic efficacy compared to the control group (12). This was a surprise as the drug combination effective for treating SARS-CoV was not effective on SARS-CoV-2 (13). These results suggest that although the M^pro^ of SARS-CoV and SARS-CoV-2 are well conserved, there is a need to identify novel inhibitors that can specifically bind to SARS-CoV-2 M^pro^.

Replication of SARS-CoV-2 depends on RdRp, and thus is also a promising drug target for the treatment of coronaviruses (6, 7). Remdesivir has shown antiviral activity against SARS-CoV-2 in vivo in rhesus monkeys through the targeting of RdRp (14). Clinical results of remdesivir in the United States showed that clinical improvement was observed in 36 out of the 53 patients (68%) with severe illness (15). In another study, double-blind, randomized trials found that COVID-19 patients taking remdesivir had an average recovery time of 11 d compared to 15 d for those taking a placebo (16). The US Food and Drug Administration (FDA) granted emergency use authorization for remdesivir to be used to treat COVID-19. On the other hand, there was a study describing that remdesivir had no statistically significant clinical benefits for severe COVID-19 patients (17). Also, the WHO recently announced that remdesivir could not reduce the hospitalization period or lower the mortality rate of COVID-19 patients. More recently, the European Medical Association announced that it should not be used for patients in intensive care units.

Computational drug repurposing is an effective approach to find new indications for the drugs already approved for other functions (Fig. 1*B*) (18). In an emergency situation like the current COVID-19 crisis, drug repurposing is a tangible strategy for developing antiviral agents within a period much shorter than that required for new drug development; the repurposed drugs will have lower failure rates as their safety in humans has already been confirmed. In general, drug repurposing starts with virtual screening of approved drugs employing various computational methods, such as molecular docking, ligand similarity, and machine learning. Virtual screening has been used to identify potential drugs against SARS-CoV-2 infection, and the results of some of such investigations have already been reported (19–23). However, the actual hit rate of virtual screening is low, and most of the predicted drug candidates are false positives. This is due to the difficulties in accurately predicting protein–ligand binding free energy. Most recently, an open-source drug discovery platform, called VirtualFlow, was used to screen more than 1.4 billion compounds through docking simulation in order to identify those chemicals binding to nuclear factor erythroid-derived 2-related factor 2 involved in inflammation through protein–protein interaction. However, the hit rate of those showing IC_50_ <60 µM was still low at ∼1.7% (24). These results suggest that it is difficult to reduce false-positive compounds with docking simulation alone, and additional strategies are required to increase the hit rates.

In this work, we developed effective filtering algorithms before and after docking simulations to improve the hit rates. In the predocking filtering process, compounds with similar shapes to the known active compounds for each target protein were selected and used for docking simulations. In the postdocking filtering process, the chemicals identified through docking simulations were evaluated, considering the docking energy and the similarity of the protein–ligand interactions with the known active compounds (Fig. 1*B* and *SI Appendix*, Fig. S1).

This virtual drug screening strategy, comprising the predocking filtering, docking simulation, and postdocking filtering processes, was applied to identify drug candidates targeting two key enzymes of SARS-CoV-2, M^pro^ and RdRp, using their crystal or cryoelectron microscopy (cryo-EM) structures recently determined (10, 19, 25). A collection of 6,218 approved and clinical trial drugs (Fig. 1*B* and *SI Appendix*, Fig. S1) was screened to rapidly discover promising repurposed drugs. After identifying drug candidates through virtual screening, cell-based SARS-CoV-2 inhibition assays were performed to select those showing efficacy. Furthermore, the combination of the best drug candidates targeting M^pro^ and RdRp as well as remdesivir was used to examine the possible synergistic efficacies in inhibiting SARS-CoV-2 with reduced toxicity.

## Results

### Structural Analysis of Coronavirus M^pro^.

An attractive drug target in coronaviruses is M^pro^ due to its essential role in processing polyproteins to produce a number of viral structural and nonstructural proteins. The crystal structure of SARS-CoV-2 M^pro^ in complex with an N3 inhibitor was first released in the Protein Data Bank (PDB 6LU7) (19). M^pro^ forms a homodimer with two protomers and comprises three domains. It processes polyproteins using a catalytic dyad consisting of His41 and Cys145 (*SI Appendix*, Fig. S2). Its active site is located between domains II and III. Each N terminus residue (N finger) binds to domain II of the other protomer, which plays an important role in the catalytic activity of M^pro^ by maintaining dimerization (26). Considering these structural characteristics, the catalytic dyad (His41 and Cys145) and N-finger binding residues (Glu166 and Phe140) were carefully analyzed for protein–ligand interactions during postdocking analysis.

As SARS-CoV-2 is closely related to other coronaviruses, including SARS-CoV and MERS-CoV, their M^pro^ enzymes showed high degrees of sequence and structure similarities (*SI Appendix*, Fig. S3). The sequence alignment showed that the M^pro^ of SARS-CoV-2 is 96% and 51% identical to those of SARS-CoV and MERS-CoV, respectively. The structure alignment also revealed that His41 and Cys145 catalytic dyad residues of M^pro^ are conserved among SARS-CoV-2, SARS-CoV, and MERS-CoV (*SI Appendix*, Fig. S3). The conserved active site of M^pro^, including the catalytic dyad and N-finger binding residues, was subjected to docking simulation of approved and clinical trial drugs.

### Virtual Screening for Identifying the Inhibitors of M^pro^.

A compound library of approved and clinical trial drugs retrieved from DrugBank (27), ZINC15 (28), and ChEMBL (29), was screened against SARS-CoV-2 M^pro^. The first task was to clean up and standardize the compounds as described in *SI Appendix*, *SI Materials and Methods*. This resulted in a curated dataset of 6,218 compounds that were used for virtual screening of drug candidates targeting the M^pro^.

The three-dimensional (3D) active ligands derived from the cocrystal structures of M^pro^ provide useful information for inferring the pharmacophore, which is a description of molecular features that are critical for molecular binding with a target protein (*SI Appendix*, Table S1). As of April 26, 2020, 103 SARS-CoV-2 M^pro^ structures (four apo forms and 99 ligand-bound forms) were deposited in the PDB. The Diamond Light Source group reported most of these structures. Although most ligands in the ligand-bound form of M^pro^ are covalent or noncovalent fragments that are relatively small and much simpler than drug-like molecules, they provide important information on examining shape and interaction similarities. Also, two structures (PDB 6LU7 and PDB 6Y2F) bound to peptidomimetic inhibitors showing inhibitory activities against SARS-CoV-2 M^pro^ were released, and the binding modes of the two inhibitors with M^pro^ showed similar patterns. The pharmacophore for M^pro^ was deduced either from a set of active ligands and the protein–ligand interactions using the structural information revealed by crystal structures. For example, a catalytic residue Cys145 frequently interacts with the heterocyclic compounds such as pyridine and pyrimidine through pi–sulfur interaction. We compared the shape and interaction similarities based on the pharmacophore to find drug candidates capable of binding to M^pro^. As a result, the above known active ligands were applied to increase the efficiency and accuracy of virtual screening (*SI Appendix*, Fig. S4).

In our virtual screening workflow for identifying drug candidates binding to M^pro^ (*SI Appendix*, Fig. S1), we started with 6,218 compounds with 1,865,400‬ 3D conformers generated by experimental-torsion basic knowledge distance geometry (ETKDG) methods (*SI Appendix*, *SI Materials and Methods*). These 3D conformers were sequentially applied to predocking ligand-based screening with shape similarity, structure-based screening through docking simulations, and postdocking screening with interaction similarity. In ligand-based screening, the shape similarity principle was used to identify potentially active compounds based on their similarities to the known active compounds (30). Twenty-five active compounds known as SARS-CoV-2 M^pro^ inhibitors in the PDB were used as template 3D conformers to identify repurposed drug candidates with similar shapes. This predocking screening process reduced the drug candidates to 4,019 compounds from the initial 6,218 compounds. The filtered 4,019 compounds were docked into the active site of the prepared structure of M^pro^ using AutoDock Vina, resulting in 398 compounds having docking energy below a threshold of −6.5 kcal/mol. This threshold was chosen to find compounds with higher binding affinity than lopinavir (docking energy, −6.0 kcal/mol), which was predicted to inhibit M^pro^ (31).

Next, postdocking simulations of examining the binding modes of the 398 hit compounds were performed based on interaction similarity with the known active compounds to identify the accurate representation of docking poses. The respective binding modes of the proposed active ligands were analyzed using 3D protein–ligand interactions using the PLIP package (32), which maps out the intermolecular interactions between the ligand and the binding pocket. The types of interactions, such as the hydrogen bonds, ionic interactions, and hydrophobic interactions with relevant amino acid residues, can be used to generate interaction similarity as Tanimoto similarity by comparing the interaction patterns of the 398 predicted hit compounds with those of the binding modes of known active ligands of SARS-CoV-2 M^pro^. After filtering out through the postdocking simulations examining interaction similarity, 15 hit compounds were obtained (*SI Appendix*, Fig. S5). Finally, these 15 compounds were manually inspected for their pharmacokinetics and side effects reported.

### Structural Analysis of Coronavirus RdRp.

The RdRp of SARS-CoV-2 comprises a nidovirus-unique N-terminal extension domain and a right-hand RdRp polymerase domain (25). The polymerase domain comprises three subdomains: a finger subdomain, a palm subdomain, and a thumb subdomain (*SI Appendix*, Fig. S2 *D* and *E*). The outer surface of RdRp has a largely negative electrostatic potential, while the RNA template and nucleotide-binding sites exhibit a strong positive electrostatic potential (*SI Appendix*, Fig. S6). The active site of RdRp is formed by the conserved polymerase motifs A-G in the finger and palm subdomains (*SI Appendix*, Fig. S2 *E* and *F*). RdRp has a central cavity surrounded by seven motifs involved in RNA template and nucleotide binding and polymerization (*SI Appendix*, Fig. S2*F*).

As replication of SARS-CoV-2 depends on RdRp, the RdRp’s of SARS-CoV-2 and other coronaviruses show high degrees of sequence and structure similarities (*SI Appendix*, Fig. S7). The sequence alignment showed that the RdRp of SARS-CoV-2 is 95% and 71% identical to those of SARS-CoV and MERS-CoV, respectively. The structure alignment also revealed that nucleotide-binding sites are highly conserved among SARS-CoV-2, SARS-CoV, and MERS-CoV (*SI Appendix*, Fig. S7). Among the nucleotide-binding sites, the conserved active site of RdRp was subjected to docking simulations of approved and clinical trial drugs.

### Virtual Screening for Identifying the Inhibitors of RdRp.

The virtual screening strategy comprising the predocking filtering, docking simulation, and postdocking filtering processes on RdRp was performed similarly to that performed on M^pro^. For RdRp, most known inhibitors such as remdesivir and favipiravir are nucleotide analogs serving as prodrugs, which are converted to their active forms having triphosphate to show therapeutic efficacy (33). Since 253 nucleotide analog compounds are deposited as inactive prodrug forms in the databases, these prodrug molecules were also converted to their active forms by automatically attaching triphosphate to ribose 5′-carbon (*SI Appendix*, Fig. S8). Then, both prodrug and active forms were subjected to docking into RdRp. In our virtual screening workflow for identifying drug candidates binding to RdRp (*SI Appendix*, Fig. S1), 1,941,300‬ 3D conformers of 6,471 compounds (6,218 compounds plus 253 active forms) were generated for shape similarity analysis. Since only one cocrystal structure of SARS-CoV-2 RdRp–ligand was available, other known active ligands (five nucleotide analogs and two nonnucleotide analogs) obtained from the RdRp–ligand cocrystal structures determined for other viruses were also used as templates for shape similarity analysis (*SI Appendix*, Table S2). Through the predocking ligand-based screening with shape similarity, the number of initial compounds was reduced to 4,554.

For docking simulation, the initial RdRp structure was obtained from the PDB (PDB 6M71) (25), which is the structure determined with 2.90-Å resolution by cryo-EM. To overcome poor resolution and consequently improve docking performance, the structure of RdRp was refined by molecular dynamics (MD) simulations to select one of populated structures with local minimum energy while having a structure similar to RdRp to which NTP is bound (*SI Appendix*, *SI Materials and Methods* and Fig. S2*F*). The 4,554 compounds filtered through predocking simulation were docked into the active site of the prepared structure of the RdRp using AutoDock Vina, resulting in 46 hit compounds with docking energy below a threshold of −6.5 kcal/mol. This threshold was set to the same threshold used for M^pro^.

Next, postdocking simulations of examining the binding modes of 46 hit compounds were analyzed based on interaction similarity with known active compounds to identify the accurate representation of docking poses (*SI Appendix*, Fig. S4). After filtering out through the postdocking simulations, 23 final hit compounds were obtained, including four nucleotide analogs and 19 nonnucleotide analogs (*SI Appendix*, Fig. S5). These final 23 compounds were manually inspected for the pharmacokinetics and side effects reported.

**Fig. 2.**
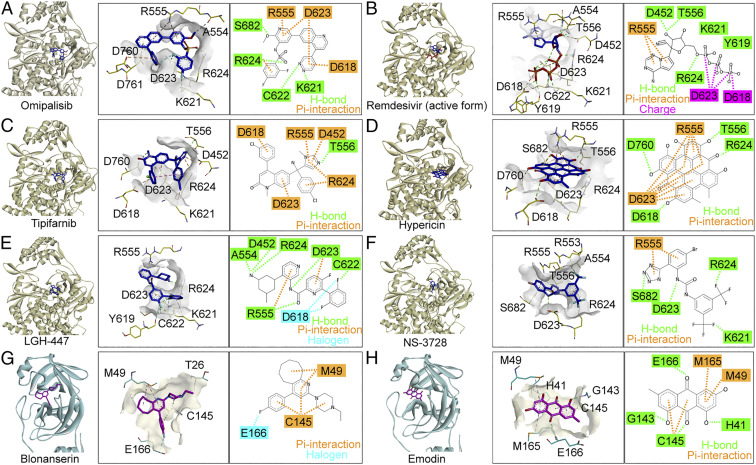
Molecular docking of drug candidates on M^pro^ and RdRp. The binding poses of six drugs (including remdesivir) with RdRp-derived structure (PDB 6M71) using AutoDock Vina: (*A*) omipalisib, (*B*) remdesivir, (*C*) tipifarnib, (*D*) hypericin, (*E*) LGH-447, and (*F*) NS-3728. The binding poses of two drugs with M^pro^ (PDB 6Y2F) using AutoDock Vina: (*G*) blonanserin and (*H*) emodin.

Our virtual screening strategy predicted at high rankings the nucleotide analog drugs that have already proven their efficacy against SARS-CoV-2; remdesivir and favipiravir show tight binding to RdRp with the docking energy of −7.1 and −6.7 kcal/mol, respectively. Remdesivir was approved by the US FDA, and the complex structure of RdRp bound with remdesivir was revealed by cryo-EM (34). It is notable that the binding mode of remdesivir predicted in our study is similar to that of the cryo-EM structure reported (Fig. 2*B*) (34). Favipiravir was also recently proven to be effective in SARS-CoV-2–infected hamsters (35) and COVID-19 patients (36).

All nucleotide analogs predicted as the final drug candidates showed binding modes similar to that of remdesivir bound to RdRp as observed by cryo-EM (34). It is notable that the structural portion corresponding to the bases of nucleotide analogs interacted with Arg555 through pi-charge or hydrogen bond interactions (*SI Appendix*, Fig. S9). The hypericin belonging to the nonnucleotide analog could most strongly bind to RdRp with the lowest binding energy of −8.9 kcal/mol through many favorable interactions such as hydrogen bond and pi-charge interactions (Fig. 2*D*).

Having identified 15 and 23 repurposed drug candidates that target M^pro^ and RdRp, respectively, we next examined their efficacies to inhibit SARS-CoV-2 in Vero cells (*SI Appendix*, Fig. S5).

### Anti-SARS-CoV-2 Activities of Single Drugs.

**Fig. 3.**
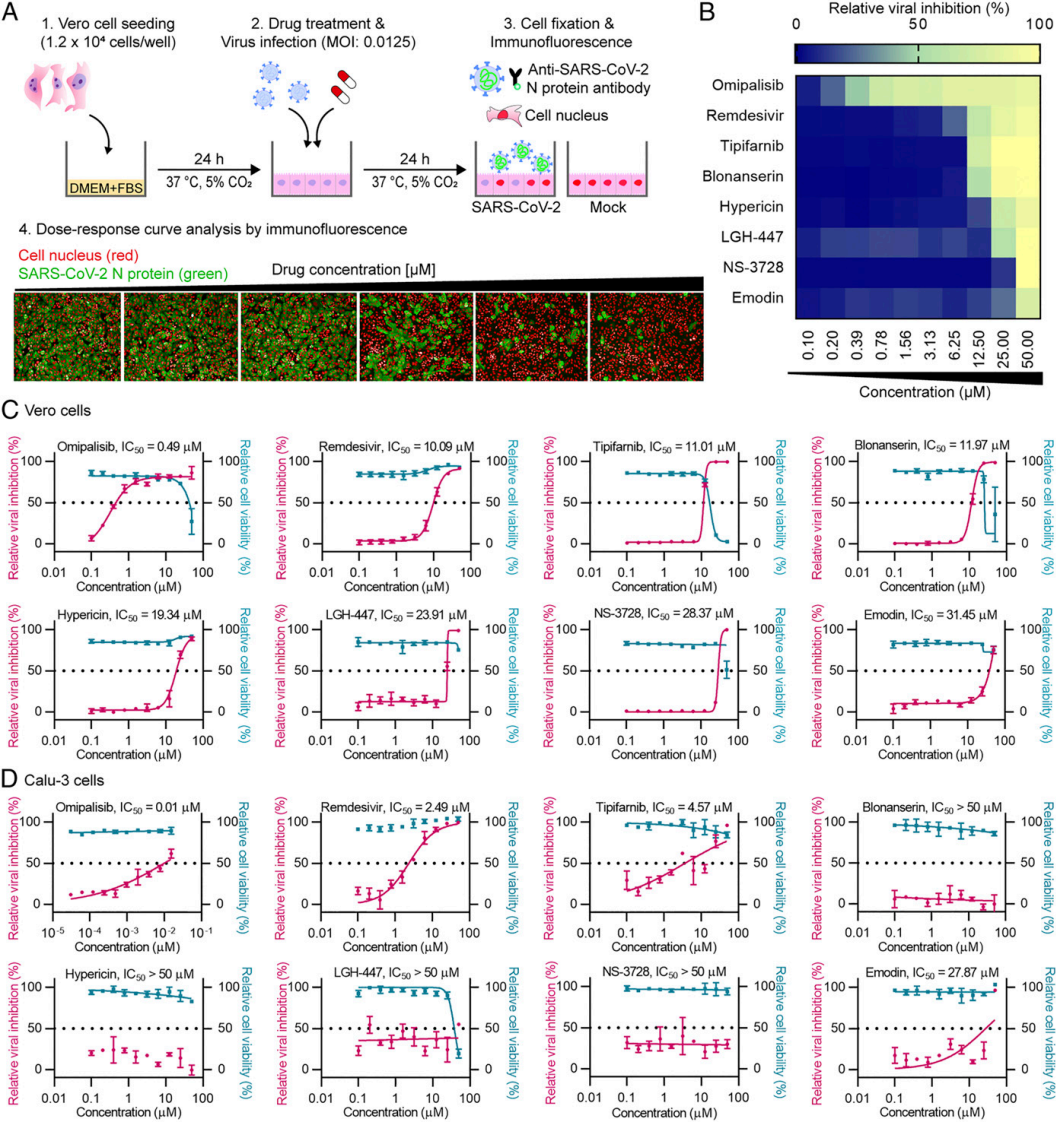
Dose–response analysis for the compounds having anti–SARS-CoV-2 activity. (*A*) A schematic of the immunofluorescence-based assay to examine anti–SARS-CoV-2 activity in Vero cells using the compounds selected from virtual screening. (*B*) A heatmap representing the percentages of normalized infection of the eight compounds in dose–response, on a scale from 0 to 100, depicting the average of duplicate independent experiments. Dose–response curves of the potent compounds in Vero cells (*C*) and Calu-3 cells (*D*). Pink line indicates relative viral inhibition and the blue line indicates relative cell viability. Data are normalized to the average of DMSO-treated wells and shown as the mean ± SD of duplicate independent experiments.

An immunofluorescence-based assay was performed to identify compounds inhibiting SARS-CoV-2 replication (Fig. 3*A*). In this assay, Vero cells were infected with SARS-CoV-2 at a multiplicity of infection (MOI) of 0.0125 immediately after administering the compounds of interest. After a 24-h incubation at 37 °C, the infected cells were scored by immunofluorescence analysis with an antibody specific for the viral N protein of SARS-CoV-2, which together with host cell nuclear staining allows quantification of the cell numbers. The confocal microscope images of both viral N proteins and cell nuclei were analyzed using our in-house Image Mining software (*SI Appendix*, *SI Materials and Methods*). Viral infection was directly quantified through immunostaining of the viral N protein. The relative viral infection was calculated by normalizing the average infection ratio of the mock control as 0% and the average infection ratio of negative control (0.5% dimethyl sulfoxide [DMSO]) as 100% in each assay plate. Recently, we used the same assay system to experimentally find niclosamide and ciclesonide as drug candidates to inhibit SARS-CoV-2 (37). Furthermore, the immunofluorescence-based assay system was validated using the known SARS-CoV-2 replication inhibitors: remdesivir, chloroquine, and lopinavir (*SI Appendix*, Fig. S10). Consistent with the results of previous in vitro cell assays against SARS-CoV-2 (38, 39), these three drugs showed antiviral activity against SARS-CoV-2 in Vero cells. Since chloroquine and lopinavir did not show efficacies in clinical trials and are no longer recommended as COVID-19 treatment (12, 40), only remdesivir was used as a reference drug to evaluate the relative antiviral activities of compounds of interest in this study.

The antiviral activity and cell cytotoxicity of 38 compounds targeting M^pro^ and RdRp identified by computational screening were examined. A dose–response curve (DRC) was generated for each compound, and three key drug performance values, the 50% inhibitory concentration (IC_50_), the 50% cytotoxicity concentration (CC_50_), and selective index (SI) defined as (CC_50_)/(IC_50_), were determined for each compound. The seven most potent and selective (SI >1) compounds against SARS-CoV-2 with IC_50_ under 50 µM (Fig. 3*C* and *SI Appendix*, Table S3) were identified; six of these compounds have never been suggested as SARS-CoV-2 inhibitors before (see below). Among them, omipalisib showed the strongest inhibition of viral replication (IC_50_ = 0.49 µM, SI = 62.84), which has 20-fold higher antiviral activity than that of remdesivir (IC_50_ = 10.09 µM, SI = 4.96). Omipalisib, also known as GSK2126458, is a highly potent inhibitor of phosphoinositide 3-kinases and mammalian target of rapamycin (41). Omipalisib has completed phase I clinical trials for the treatment of solid tumors (42) and idiopathic pulmonary fibrosis (IPF) (43). COVID-19 causes a wide range of respiratory diseases, one being IPF (44). Thus, omipalisib might provide dual benefits of antiviral and antifibrotic efficacies in COVID-19 patients. More recently, an independent study suggested a different mechanism of omipalisib exhibiting anti–SARS-CoV-2 activity in human cells; omipalisib inhibited growth factor receptor signaling of host cell, which is activated in response to viral infection (45). This result together with ours suggest that omipalisib might be able to simultaneously inhibit viral and host protein targets, resulting in higher antiviral activity.

Other than omipalisib among seven potent compounds, two of them (tipifarnib, IC_50_ of 11.01 µM; blonanserin, IC_50_ of 11.97 µM) showed efficacies similar to remdesivir (IC_50_ of 10.09 µM). Tipifarnib is a potent farnesyltransferase inhibitor for treating cancers such as pancreatic neoplasms and acute myeloid leukemia, which has completed phase III clinical trial (46). Blonanserin is an antipsychotic drug approved in Japan and Korea for the treatment of schizophrenia (47). The other four compounds, hypericin, LGH-447, NS-3728, and emodin, did not surpass the potency of remdesivir.

We performed a fluorescence resonance energy transfer (FRET) assay for the two drugs (emodin and blonanserin) against M^pro^. The results showed that both drugs showed inhibitory activities against M^pro^. As controls, GC-376, a positive control, showed inhibitory activity against M^pro^, while omipalisib, a negative control drug effective against RdRp, showed no inhibitory activity against M^pro^ (*SI Appendix*, Fig. S11). Thus, the computational simulation strategy developed here successfully predicted two drugs (emodin and blonanserin) effective against M^pro^. For FRET assay of RdRp, we were not able to show the activities of the drugs we identified using the SARS-CoV-2 RdRp assay kit (*SI Appendix*, *SI Materials and Methods*). Thus, we alternatively investigated protein–drug binding capability by calculating the binding free energy from the molecular mechanics Poisson–Boltzmann surface area (MM/PBSA) method using MD trajectories. The calculated binding free energies of remdesivir, omipalisib, tipifarnib, NS-3728, hypericin, and LGH-447 for the RdRp of SARS-CoV-2 were −231.142, −207.985, −200.967, −199.029, −196.670, and −180.234 kJ/mol, respectively, indicating that five drugs had binding free energies similar to that of remdesivir (*SI Appendix*, Table S4). Also, the results of MD simulation were analyzed based on the rmsd to examine the stability of the protein–drug complex (*SI Appendix*, Fig. S12). All the results of rmsd for six drugs maintained an average range distance between 0.1 and 0.3 nm. Thus, it could be concluded that the protein–drug complexes were stable. Considering the MD simulations analysis based on rmsd and binding free energy by MM/PBSA, five drugs identified in this study can bind to the active site of RdRp and inhibit viral RNA synthesis.

Next, seven potent drugs showing anti–SARS-CoV-2 activities in Vero cells were also tested in human lung epithelial Calu-3 cells. Among them, three drugs, emodin, omipalisib, and tipifarnib, showed antiviral activities against SARS-CoV-2 (Fig. 3*D*). Interestingly, all three drug candidates showed higher antiviral activities in Calu-3 cells compared with Vero cells. In particular, omipalisib showed antiviral activity against SARS-CoV-2 in Calu-3 cells with the IC_50_ value that was 49-fold lower than that observed in Vero cells (IC_50_ of 10.04 nM in Calu-3 cells and IC_50_ of 0.49 µM in Vero cells). Also, tipifarnib showed an IC_50_ of 4.57 µM, which was much lower than that in Vero cells (11.01 µM). Likewise, the IC_50_ values of emodin in Vero and Calu-3 cells were 31.45 and 27.87 µM, respectively. Thus, the three drug candidates showed greater potential in treating SARS-CoV-2 in human.

It is notable that the IC_50_ values of most compounds (all except for NS-3728) we found effective against SARS-CoV-2 in Vero cells were greater than the reported maximum serum concentration (C_max_) values in human cells (*SI Appendix*, Table S3). Omipalisib showed the IC_50_ value close to, yet slightly greater than, the C_max_ value. Thus, a strategy of providing antiviral activity at a concentration below the C_max_ is needed. One such strategy is the use of drug combinations, through which synergistic efficacy might be achieved at lower concentration of each drug, and also at reduced toxicity. Thus, we next examined the combinations of drug compounds identified through virtual screening together with remdesivir for their possible synergistic efficacy.

### Drug Combinations Showing Synergistic Antiviral Activities.

Combination therapies have the potential to increase efficacy of treatment while reducing effective concentration of individual compound below the maximal plasma concentration, possibly reducing the toxicity of each drug observed at higher concentration if used alone. Also, the FDA-approved remdesivir showed limited efficacy, which might be improved by combination therapy. Furthermore, drug candidates we identified are targeting M^pro^ (blonanserin and emodin) and RdRp (omipalisib, tipifarnib, hypericin, LGH-447, and NS-3728), and thus combinations of drugs each targeting M^pro^ and RdRp might enhance SARS-CoV-2 inhibition by simultaneously blocking two key proteins in the virus. To investigate such synergistic efficacies, 10 drug combinations comprising five compounds (remdesivir, blonanserin and emodin targeting M^pro^, and omipalisib and tipifarnib targeting RdRp) were designed. These drug combinations were evaluated using a checkerboard assay with a twofold serial dilution from 4×IC_50_, where the IC_50_ values were determined from separate single-drug experiments described above.

The synergistic antiviral activities of drug combinations were evaluated by calculating the instantaneous inhibitory potential (IIP) (48) (*SI Appendix*, Fig. S13 and Note S1). IIP reflects the log reduction in viral replication at a clinically relevant drug concentration through the DRC slope. The synergistic effects of combined drugs can be assessed by comparing the experimentally determined IIP with two IIP values calculated using two methods (49) that consider competitive binding (Loewe additivity) (50) and independent inhibition (Bliss independence) (51), which represent the degrees of antagonistic and synergistic interactions, respectively. The degree of independence (DI) (52, 53) which is (experimental IIP minus Loewe additivity IIP) divided by (Bliss independence IIP minus Loewe additivity IIP), was calculated (*SI Appendix*, Fig. S13 and Note S2). In general, the DI value becomes close to 1 when the drug combination shows independent inhibition (e.g., experimental IIP close to Bliss independence IIP), while it becomes close to 0 when the drug combination shows competitive binding (e.g., experimental IIP close to Loewe additivity IIP). Thus, the DI value becomes higher when the drug combination shows synergy and can become greater than 1 depending on the experimental IIP value. Based on the DI values, the drug combination effect can fall into five categories: synergy, Bliss, intermediate, Loewe, and antagonism (52) (*SI Appendix*, Fig. S13 *A* and *B*).

The DI values of 10 drug combinations were calculated (*SI Appendix*, Fig. S13*B*). First, in order to determine the maximum concentration of each drug for combination therapy considering cytotoxicity, drugs were combined at various concentrations from their initial concentrations of 0.125×IC_50_ to 2×IC_50_. It was found that each drug combination given at 1×IC_50_ sufficiently inhibited SARS-CoV-2 replication without cytotoxicity. Among the 10 drug combinations tested, 6 drug combinations showed synergistic effects (*SI Appendix*, Fig. S13*B*). The threshold of DI for synergy evaluation was set at 1.2 as in a previous study (52). Synergistic effects significantly greater than Bliss were observed when the combinations of M^pro^ with RdRp inhibitors were used (*SI Appendix*, Fig. S13 *C* and *D*); the DI values were 2.68 and 2.22 when tipifarnib/blonanserin combination and emodin/remdesivir combination, respectively, were used. These results suggest that synergistic effects can be maximized when different drug targets are independently inhibited.

**Fig. 4.**
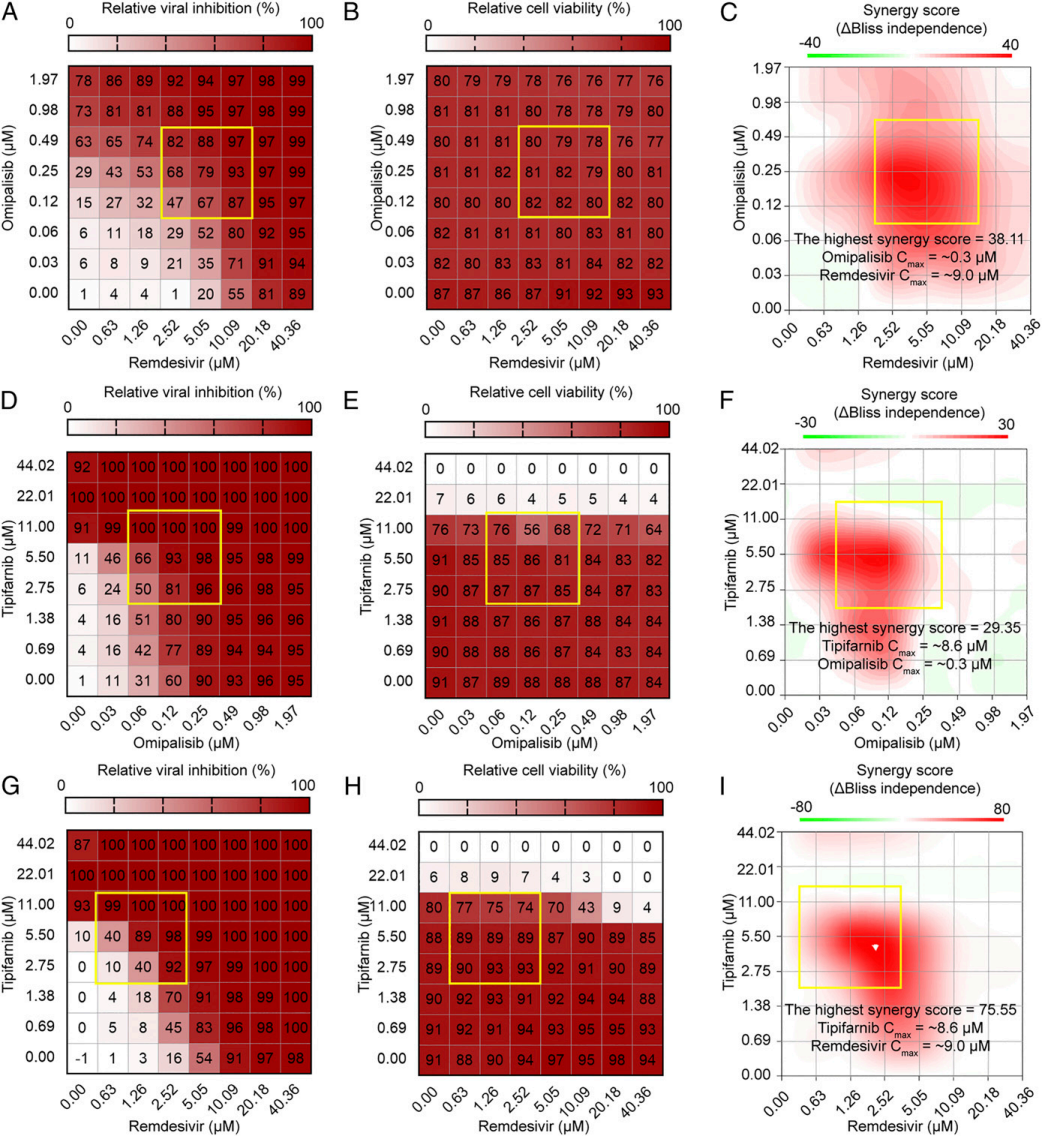
Analyses of drug combinations on anti–SARS-CoV-2 activity, cell viability, and their synergistic effects. Two-dimensional matrix of dose–response for relative viral inhibition: (*A*) omipalisib/remdesivir, (*D*) tipifarnib/omipalisib, and (*G*) tipifarnib/remdesivir. The heatmap depicts relative viral inhibition scaled to the range of 0 to 100%. Two-dimensional matrix of dose–response for relative cell viability: (*B*) omipalisib/remdesivir, (*E*) tipifarnib/omipalisib, and (*H*) tipifarnib/remdesivir. The heatmap depicts relative cell viability scaled to the range of 0 to 100%. Topographic two-dimensional map of synergy scores determined by synergyfinder using the data in *A*, *D*, and *G*, respectively: (*C*) omipalisib/remdesivir, (*F*) tipifarnib/omipalisib, and (*I*) tipifarnib/remdesivir. The synergy map highlights synergistic and antagonistic dose regions in red and green colors, respectively. A yellow box represents the area with the highest synergy score obtained by synergyfinder.

Contrary to in vitro studies, there are a number of factors that can influence clinical efficacy. For example, if the therapeutic dose causes severe adverse effects in a patient, the drugs shown to be effective in vitro cannot have clinical utility. If the drug does not achieve an effective serum concentration in a patient, or if IC_50_ is significantly greater than the achievable C_max_, then the drug is unlikely to have therapeutic utility either. To investigate the optimal concentration of the drug combination considering cytotoxicity and C_max_, the synergyfinder R package was used to assess the effects of drug combinations using the Bliss independence model, where a Δscore > 10 indicates likely synergy, Δscore < −10 indicates antagonism, and Δscore between −10 and 10 suggests an additive interaction (54). The synergy scores can be interpreted as the average excess response due to drug interactions (i.e., Δscore of 75 corresponds to 75% of response beyond expectation). For the three drug combinations (omipalisib/remdesivir, tipifarnib/omipalisib, and tipifarnib/remdesivir), strong synergistic effects were confirmed within the C_max_, and noncytotoxic concentrations with synergy scores ranged from 30 to 70 (Fig. 4). Similar results were obtained for synergy analysis using the zero interaction potency (ZIP) model (*SI Appendix*, Figs. S14–S23). For omipalisib/remdesivir, remdesivir at 5.05 µM in combination with omipalisib at 0.25 µM showed about 79% viral inhibition with a synergy score of 38.11, which can reduce the dose of each drug while maintaining the higher antiviral activity. The synergistic effect of tipifarnib was only analyzed at lower than 11 µM, due to the cytotoxicity of tipifarnib at higher concentration. For tipifarnib/remdesivir, remdesivir at 1.26 µM in combination with tipifarnib at 5.50 µM achieved 89% viral inhibition without significant cytotoxicity. These results suggested that drug combinations can increase the efficacy of COVID-19 treatment even at lower therapeutic doses of each drug below C_max_, which consequently provides opportunities of reducing toxicity of each drug.

## Discussion

Although a number of institutions and companies around the world are racing to develop small molecule drugs to treat COVID-19, an effective one has not yet been developed. Although the FDA approved remdesivir for treating COVID-19 patients (16), the high mortality despite the use of remdesivir (16) and recent recommendations by the WHO and European Medical Association suggested that treatment with remdesivir alone is not effective. Furthermore, we should be prepared for the emergence of SARS-CoV-2 variants and other pathogenic viruses we never encountered before by establishing strategies for rapidly developing therapeutic drugs along with vaccines.

In this study, we aimed at developing strategies for rapidly identifying drug candidates by drug repurposing through virtual screening algorithms comprising predocking simulation, docking simulation, and postdocking simulation. Virtual screening of 6,218 FDA-approved and clinical trial drug compounds, 15 and 23 potential compounds targeting M^pro^ and RdRp, respectively, could be identified. The above virtual screening of 6,218 compounds targeting M^pro^ and RdRp took about 2.5 d using a workstation having central processing units (CPUs) with 32 cores and 64 threads using the computer system described *SI Appendix*, *SI Materials and Methods*, Computation Environment; for one compound, the average CPU time was ∼949 s to run predocking, docking, and postdocking simulations. Thus, the virtual screening algorithms reported here allow rapid identification of drug candidates for the given target proteins.

These compounds identified by virtual screening were subjected to immunofluorescence-based assays to find potent antiviral drugs against SARS-CoV-2 in Vero cells (Fig. 3 *B*–*D*). Notably, 7 out of 38 compounds showed efficacies in inhibiting SARS-CoV-2, corresponding to a hit rate of 18.4%. This hit rate is significantly higher than the hit rate of about 1 ∼ 2% obtainable by typical virtual screening on new targets. Among the 7 compounds showing SARS-CoV-2 inhibition, omipalisib showed the highest potency and selective index against SARS-CoV-2. As described earlier, omipalisib has an advantage of inhibiting SARS-CoV-2 replication by binding to RdRp and also inhibiting growth factor receptor signaling of human cells (45). Furthermore, omipalisib has completed phase I clinical trials as an indication for IPF, so it could prevent or treat pulmonary fibrosis, a severe life-threatening sequela caused by SARS-CoV-2 infection. Also, we performed additional docking simulations of seven drugs for the RdRp and M^pro^ of SARS-CoV and MERS-CoV (*SI Appendix*, Fig. S24). All seven drugs showed similar binding modes and binding affinities to their respective drug targets of coronaviruses. As SARS-CoV-2 is closely related to other coronaviruses, their M^pro^ and RdRp showed high degrees of sequence and structure similarities. These results suggest that the drug candidates identified in this study may also be effective in inhibiting SARS-CoV and MERS-CoV.

We evaluated the efficacies of the seven drugs identified in Vero cells for anti–SARS-CoV-2 activity in human lung epithelial Calu-3 cells as well. Among them, three drugs showed antiviral activities against SARS-CoV-2. Interestingly, all three drug candidates showed higher antiviral activities in Calu-3 cells compared with Vero cells. These data suggest that there are obviously different cellular mechanisms of viral infection in different cell types of different organisms, suggesting the importance of drug efficacy testing in human lung epithelial cells in this case.

As mentioned above, the IC_50_ values of the six (out of seven) compounds we found effective against SARS-CoV-2 in Vero cells were greater than the reported C_max_ values in human cells (*SI Appendix*, Table S3). This problem was overcome by taking the drug combination approach, which demonstrated higher antiviral activity even at concentrations below the C_max_ (Fig. 4). Such drug combination therapy also reduces the risk of each drug’s toxicity at its higher concentration. Through this study, at least three promising drug combinations (omipalisib/remdesivir, tipifarnib/omipalisib, and tipifarnib/remdesivir) emerged with high efficacy of SARS-CoV-2 inhibition at their clinically achievable concentrations.

Based on the promising results obtained with omipalisib and three drug combinations in cell-based SARS-CoV-2 inhibition assays, we plan to move forward to preclinical and clinical trials. Although we used drug repurposing of 6,218 drug compounds as an example for rapidly identifying SARS-CoV-2 inhibitor drugs through the application of our virtual screening workflow, any compound library can be employed in the screening process. Taken together, the virtual screening strategy we reported here will be useful for rapid identification of drug candidates for any known target protein, whether it is of viral, prokaryotic, or eukaryotic origin, as long as the cocrystal structures of the target protein bound to ligands are available.

## Materials and Methods

All the materials and methods conducted in this study are detailed in *SI Appendix*, *SI Materials and Methods*: structure preparation of M^pro^; structure preparation of RdRp; compound library preparation; predocking filtering with shape similarity; molecular docking simulations; postdocking filtering with interaction similarity; virus and cells; reagents; immunofluorescence assay of SARS-CoV-2 infection; M^pro^ and RdRp assays; binding free energy calculation; and computation environment.

## Supplementary Material

Supplementary File

## Data Availability

Source code for the virtual screening of drug repurposing against SARS-CoV-2 is available at https://bitbucket.org/kaistsystemsbiology/vs-covid19 (55). All data supporting the findings presented in this study are available in the main text and *SI Appendix*.
