# Supplementary material for: Drugs repurposed for COVID-19 by virtual screening of 6,218 drugs and cell-based assay
Source: Proc Natl Acad Sci U S A. 2021 Jul 7;118(30):e2024302118. doi: 10.1073/pnas.2024302118 (PMC8325362; doi:10.1073/pnas.2024302118)
Supplement: Supplementary File [file pnas.2024302118.sapp.pdf]

PNAS
www.pnas.org

Supplementary Information for

Drugs repurposed for COVID-19 by virtual screening of 6,218 drugs and cell-based assay

Woo Dae Jang, Sangeun Jeon, Seungtaek Kim, Sang Yup Lee

Correspondence should be addressed to: Sang Yup Lee or Seungtaek Kim
Email: leesy@kaist.ac.kr or seungtaek.kim@ip-korea.org

This PDF file includes:

Materials and Methods
Figures S1 to S24
Tables S1 to S4
Notes S1 to S2
SI References

Materials and Methods

Structure preparation of M^{pro}. The crystal structure of SARS-CoV-2 M^{pro} was obtained from the PDB (PDB 6Y2F) (1). The structure was solved with a 1.95Å resolution in complex with an α-ketoamide inhibitor. Prior to molecular docking, two missing residues (Glu47 and Asp48) of the loop were grafted from another M^{pro} crystal structure (PDB 6LU7) (2), followed by performing energy minimization using the steepest descent methods using GROMACS 4.5 package (3). In addition, all solvent molecules and the ligand were removed.

Structure preparation of RdRp. The RdRp of SARS-CoV-2 was obtained from the PDB (PDB 6M71) (4). This structure was solved at 2.90Å resolution by cryo-EM. To overcome low resolution and improve docking performance, the final structure was refined by molecular dynamics (MD) simulations using GROMACS 4.5 package (3). During the preparation procedure for the MD simulation, the CHARMM27 all-atom force field (5) was used to generate the protein topology and the TIP3P water model (6). The RdRp structure was solvated into a dodecahedron box under periodic boundary conditions. To neutralize the system, counter ions were added accordingly. The steepest descent energy minimization stabilizes the protein by removing possible unfavorable interactions until the energy has converged below 850 kJ/mol. Following minimization, the system was equilibrated in two steps with position restraints on the heavy atoms of the protein. The first equilibration phase was conducted under NVT (canonical ensemble) for 100 ps at 300 K. Next, equilibration of pressure was performed under NPT (isothermal-isobaric ensemble) for 200 ps. Finally, a production MD simulation was performed in the absence of any restraints. Short-range non-bonded interactions were cutoff at 1.2 nm with long-range electrostatics calculated using the particle-mesh Ewald (PME) (7). The product simulation at 300 K and 1 bar was continued for 5 ns. The accumulation of coordinates from 2 ns to 5 ns was set to every 1ps. Based on the root mean-square deviation (RMSD) comparison of each snapshot from the MD simulation, clusters were generated by controlling the cutoff value as 0.12 nm using the gromos method implemented in GROMACS 4.5 package (g_cluster). Based on structural clustering analysis, the structure of RdRp for docking analysis was refined to select one of populated structures with local minimum energy by referring to the RdRp structure of norovirus (PDB 3H5Y (8); resolution: 1.77Å) to which NTP was bound; we used the co-crystal structure of RdRp-NTP of norovirus because that of SARS-CoV-2 was not available when our simulations were performed. Thus, the same procedure can be taken using the co-crystal structure of RdRp-NTP (PDB 7BV2 (9); resolution: 2.50Å) of SARS-CoV-2 recently published.

Compound library preparation. Non-redundant 6,218 approved and clinical trial drugs from the DrugBank (10), ZINC15 (11), and ChEMBL (12) were collected and preprocessed for virtual screening to discover repurposed drugs. For DrugBank, all compounds categorized as approved and investigated drugs were downloaded, and in the case of ZINC15, all compounds categorized as in-trials drugs were downloaded. The compounds from the ChEMBL were downloaded using the following activity keywords: “polymerase”, “protease”, and “antiviral” with max clinical phase I to IV. All compounds were further subjected to standardization procedure and the removal of salts using the MolVS (https://molvs.readthedocs.io/en/latest/) implemented in RDKit (13). The duplicate structures were removed by InChI collisions. When preparing compound library for RdRp, nucleotide analog pro-drugs were converted to their active forms by automatically attaching triphosphate to ribose 5’-carbon using the customized reaction rules (*SI Appendix*, Fig S7). We created corresponding reaction rules using metabolic reactions of phosphorylation of ribose in nucleotide analog drugs, such as remdesivir and favipiravir, using the web-based RetroRules (https://retrorules.org) (14). Four reaction rules were constructed using known nucleotide analog pro-drugs (remdesivir, favipiravir, galidesivir, and EIDD-2801). These reaction rules are described in SMiles ARbitrary Target Specification (SMARTS), a chemical language describing structural patterns of molecules (*SI Appendix*, Fig S7). The reactor algorithm implemented in RDKit (13) takes constructed reaction rules and a pro-drug molecule as inputs, which are presented in SMARTS and simplified molecular-input line-entry system (SMILES), respectively, to generate active forms of pro-drugs.

Pre-docking filtering with shape similarity. The active ligands for M^{pro} and RdRp obtained from the co-crystal structures of SARS-CoV-2 or other viruses were used as a template for pre-docking screening with shape similarity (*SI Appendix*, Tables S1 and S2). On the other hand, since compounds of ligand library prepared ourselves do not contain 3D structural information, 3D conformers of all compounds were generated using the ETKDG method (15) implemented in RDKit

package (13). The values of ligand shape similarity between the template compounds and the compounds of in-house ligand library were calculated using three methods, including Ultrafast Shape Recognition (USR) (16), USR with Credo Atom Types (17), ElectroShape (18). Three shape similarity methods were performed using open drug discovery toolkit (https://oddt.readthedocs.io/en/latest/) (19). The average values of the three methods were finally used to evaluate the 3D shape similarity. The shape similarity threshold was set to 0.4. The resulting compounds obtained through shape similarity screening were subjected to perform docking simulations in the next step.

Molecular docking simulations. AutoDock Vina (v1.1.2) (20) was used for docking simulations to evaluate binding affinity between 6,218 approved and clinical trial drugs and target proteins (M^{pro}, RdRp). PDBQT format, an input for the AutoDock Vina, was prepared using OpenBabel (v2.4.1) (21) and MGLTools (v1.5.6) to convert SDF to PDBQT. Dimension of the grid box used for docking was set to 12Å, 12Å, and 12Å in x, y, and z direction, respectively. The grid box centers were defined as the center of a native ligand bound to the target protein (M^{pro}, PDB 6Y2F; RdRp, PDB 3N6M). The exhaustiveness parameter that controls the extent of the search was set to 8. For each ligand, up to 10 binding modes were generated with an energy range of 4 kcal/mol. Finally, virtual hit compounds were selected whose computed docking energy was less than or equal to cutoff energy (M^{pro}, -6.5 kcal/mol; RdRp, -6.5 kcal/mol). Protein structures were visualized using Discovery Studio Visualizer (v16.1.0.15350). These were subsequently employed in the next round of screening incorporating interaction similarity.

Post-docking filtering with interaction similarity. Post-docking simulations were performed based on protein-ligand interaction similarity with the known active compounds to identify the accurate representation of docking poses. As such, the protein-ligand interactions of the binding poses obtained by the docking were analyzed with PLIP package (v1.4.5) (22). It returns a list of detected interactions between each compound and the amino acids of the target receptor, covering six interaction types (hydrogen bonds, hydrophobic contacts, pi-stacking, pi-cation interactions, salt bridges, and halogen bonds). The types of interactions with relevant amino acid residues can be used to generate interaction similarity as Tanimoto similarity by comparing the interaction patterns of the predicted hit compounds with those of the binding modes of known active ligands of the target proteins. The interaction similarity threshold was set to 0.3.

Virus and cells. Vero cells were obtained from the American Type Culture Collection (ATCC CCL-81) and maintained at 37°C with 5% CO_2 in Dulbecco's modified eagle medium (DMEM; Welgene), supplemented with 10% heat-inactivated fetal bovine serum (FBS) and 1× antibiotic-antimycotic solution (Gibco). For Calu-3, cells were seeded at 2.0×10^4 cells per well in Eagle's minimum essential medium (EMEM), supplemented with 20% FBS, 1× MEM Non-Essential amino acid and 1× antibiotic-antimycotic solution (Gibco) in black, 384-well, μClear plates (Greiner Bio-One), 24 h prior to the experiment. SARS-CoV-2 (βCoV/KOR/KCDC03/2020) was provided by Korea Centers for Disease Control and Prevention (KCDC) and was propagated in Vero cells. All experiments involving live SARS-CoV-2 followed the guidelines of the Korea National Institute of Health (KNIH) using enhanced biosafety level 3 (BSL3) containment procedures at Institut Pasteur Korea approved for use by the KCDC.

Reagents. Remdesivir (HY-104077) was purchased from MedChemExpress (Monmouth Junction, NJ). Blonanserin, emodin, hypericin, omipalisib, and tipifarnib were purchased from LEAP Chem Co., Ltd. (Hangzhou, China). NS-3728 was purchased from J&H Chemical Co., Ltd. (Hangzhou, China). LGH-447 was purchased from Chemme Co., Ltd. (Hangzhou, China). All reagents were dissolved in dimethyl sulfoxide (DMSO) for the screening. Anti-SARS-CoV-2 N protein antibody was purchased from Sino Biological Inc. (Beijing, China). Alexa Fluor 488 goat anti-rabbit IgG (H+L) secondary antibody and Hoechst 33342 were purchased from Molecular Probes. Paraformaldehyde (PFA) (32% aqueous solution) and normal goat serum were purchased from Electron Microscopy Sciences (Hatfield, PA) and Vector Laboratories, Inc. (Burlingame, CA), respectively.

Immunofluorescence assay of SARS-CoV-2 infection. Infected Vero and Calu-3 cells were subjected to evaluation of antiviral activity using an immunofluorescence-based imaging assay, labeling viral N protein of the SARS-CoV-2 within infected cells. In each assay detailed below, including dose-response assays and drug synergy assays, Vero cells were seeded at 1.2×10^4 cells per well in DMEM, supplemented with 2% FBS and 1× antibiotic-antimycotic solution (Gibco), in black,

384-well µClear plates (Greiner Bio-One) 24 h prior to the experiment. Ten-point DRCs were generated, with compound concentrations ranging from 0.1 to 50 µM. For the viral infections, plates were transferred into the BSL3 containment facility, and SARS-CoV-2 was added at multiplicity of infection (MOI) of 0.0125. Before validation experiments with Vero cells, we examined both cell viability and cell infectivity by changing the MOI of SARS-CoV-2. MOI of 0.0125 was chosen as the best experimental condition based on the best cell viability (91.73%) and the highest virus infectivity (75.75%). For Calu-3, cells were seeded at 2.0 × 10^4 cells per well in EMEM, supplemented with 20% FBS, 1× MEM Non-Essential amino acid and 1× antibiotic-antimycotic solution (Gibco) in black, 384-well, µClear plates (Greiner Bio-One), 24 h prior to the experiment. Ten-point DRCs were generated with compound concentrations ranging from 0.1 to 50 µM. Omipalisib was analyzed at concentrations ranging from 0.031 to 15.63 nM due to cytotoxicity. For viral infection, plates were transferred into the BSL-3 containment facility, and SARS-CoV-2 was added at MOI of 0.5. The Vero and Calu-3 cells were fixed at 24 hours post-infection with 4% PFA and permeabilized with Triton-X100 to promote entering antibodies into cells. The acquired images were analyzed using in-house software to quantify cell numbers and infection ratios, and antiviral activity was normalized to positive (mock) and negative (0.5% DMSO) controls in each assay plate. DRCs were fitted by sigmoidal dose-response models, with the following equation: $Y = \text{bottom} + (\text{top} - \text{bottom})/[1 + (IC_{50}/X)^{\text{Hillslope}}]$, using Prism7. IC_{50} values were calculated from the normalized activity dataset-fitted curves. All IC_{50} and CC_{50} values were measured in duplicate, and the quality of each assay was controlled by Z'-factor and the coefficient of variation in percent (%CV). For drug synergy quantification, drug combinations were evaluated using a checkerboard assay at eight points with a 2-fold serial dilution from 4 × IC_{50}, where the IC_{50} values were determined in separate single-drug experiments. Synergy analysis was performed using synergyfinder R-package (v2.4.0) (23) using Bliss independence and ZIP models.

M^{pro} and RdRp assays. The M^{pro} enzyme assay was performed in black, 384-well, µClear plates (Greiner Bio-One) with a total volume (25 µL). Inhibition of enzyme activity was evaluated using 3CL protease, untagged (SARS-CoV-2) assay kit obtained from BPS Bioscience, Inc. (Catalog number, 100823; San Diego, USA) with compounds concentrations ranging from 0.1 to 50 µM according to the manufacturer's instructions. Fluorescent intensity was measured on Ensight™ Multimode Microplate Reader (PerKinElmer, Inc.). The results were normalized to positive control in each assay plate. DRCs were fitted by sigmoidal dose-response models, with the following equation: $Y = \text{bottom} + (\text{top} - \text{bottom})/[1 + (IC_{50}/X)^{\text{Hillslope}}]$, using Prism7. IC_{50} values were calculated from the normalized activity dataset-fitted curves.

For RdRp assay, we used a commercially available kit (Catalog number, S2RPA020KE; Lot number, 170201008) from ProFoldin Inc. (Hudson, MA, USA) and SARS-CoV-2 RdRp enzyme (Catalog number, 100839; Lot number, 201123) from BPS Bioscience, Inc. (San Diego, CA, USA). However, even remdesivir triphosphate (RDV-TP) as a positive control drug did not show activity even at high concentrations (50 µM), which suggests that there is a problem with the enzyme assay kit; Profoldin's RdRp enzyme assay kit was the only available kit at the time of our revision. Thus, we instead performed additional binding free energy calculation.

Binding free energy calculation. The protein-ligand complexes derived from docking simulations were subjected to MD simulations by GROMACS 4.5 package (3). The CHARMM36 force field was assigned to the protein. Ligand parameterization was performed using CHARMM General Force Field (24). Each system was immersed in a dodecahedron box of TIP3P water. The Na^+ or Cl^- was applied to neutralize the system. The systems were first minimized using the steepest descent methods. After minimization, the systems were heated from 0 to 300 K over 100 ps using the NVT ensemble with a weak restraint on the enzyme and ligand. Following this, the systems were equilibrated over 200 ps at a constant pressure of 1 bar and temperature of 300 K using the NPT ensemble. Finally, the 3 ns production run was performed. Based on the 3 ns MD trajectory, binding free energy was calculated with molecular mechanics Poisson-Boltzmann surface area (MM/PBSA). The MM/PBSA calculations were performed using g_mmpbsa (25). The binding free energy was calculated according to the following equation: $\Delta G_{cal} = \Delta H - T\Delta S = \Delta E_{vdw} + \Delta E_{ele} + \Delta G_{pb} + \Delta G_{np} - T\Delta S$, where ΔE_{vdw} and ΔE_{ele} refer to van der Waals energy and electrostatic terms, respectively. ΔG_{pb} and ΔG_{np} refer to polar and nonpolar solvation free energies, respectively. The entropy term ($T\Delta S$) was not calculated in this study. Since the multiple ligands were compared based on the same target, it is reasonable to ignore the entropy.

Computation environment. All the locally installable software programs or scripts were implemented in an automatic fashion using Python 3.6 under Linux Ubuntu 16.04. All simulations were performed on a workstation (Intel® Xeon® Gold 6130 2.10 GHz CPUs with 32 cores and 64 physical threads in total and a 256 GB RAM)

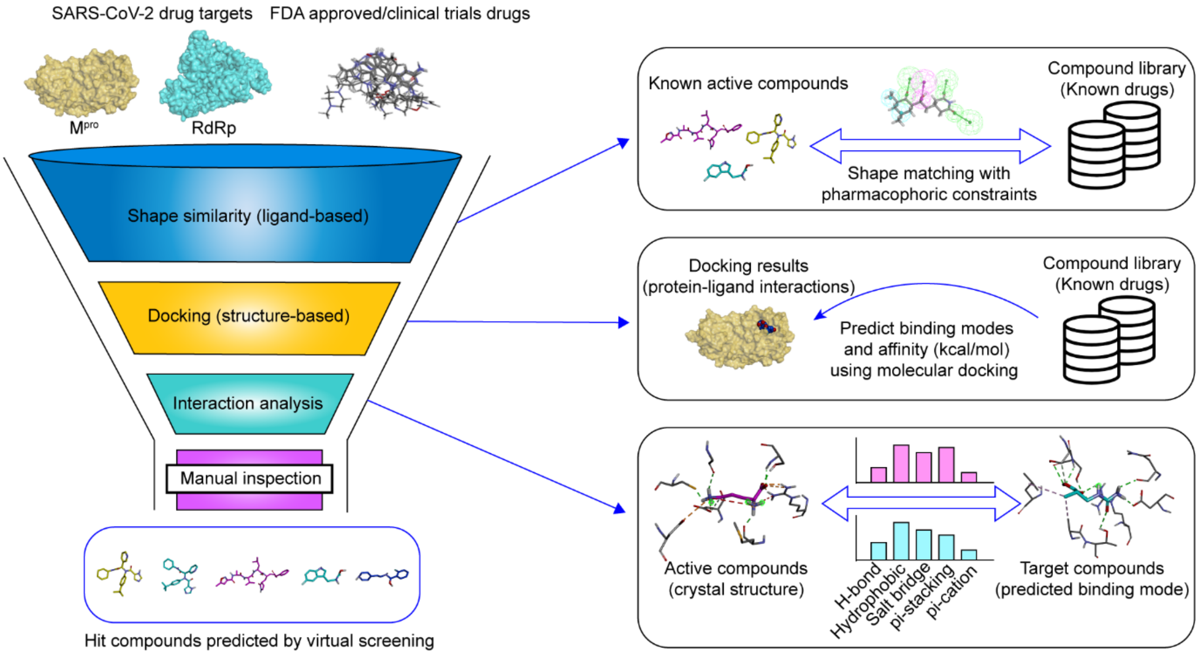

Fig. S1. Virtual screening strategy of combined pre-docking, docking, and post-docking simulations. To reduce false positives often obtained by performing docking simulation alone, pre-docking and post-docking simulations were performed to filter drug candidates. In the pre-docking filtering process, compounds with similar shapes to the known active compounds for each target protein were selected. In the post-docking filtering process, the chemicals identified through docking simulations were evaluated considering the docking energy and the similarity of the protein-ligand interactions with the known active compounds.

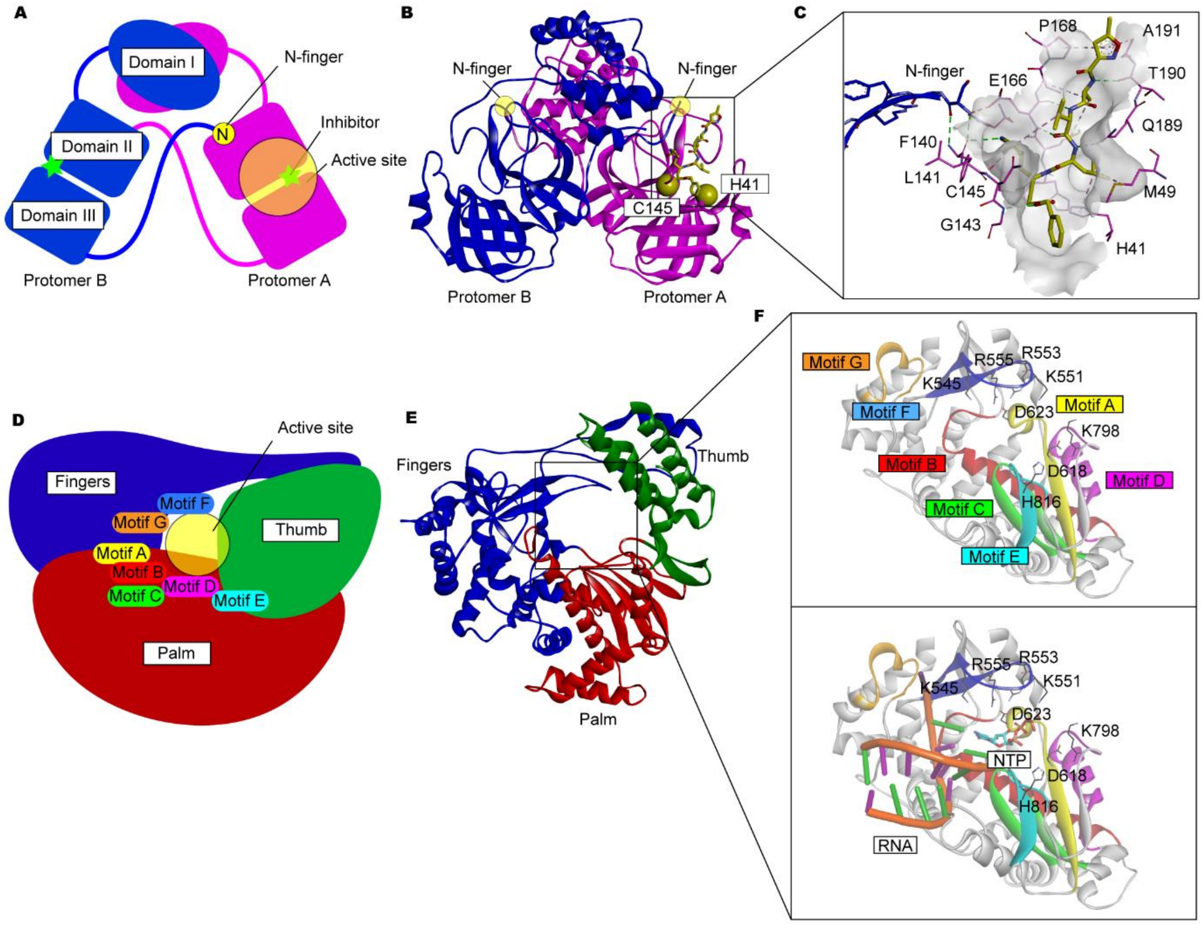

Fig. S2. The structures of M^{pro} and RdRp of SARS-CoV-2. (A) Cartoon diagram of M^{pro} representing three domains and showing an important role of N-finger in dimerization. (B) Homodimer of M^{pro} in complex with an N3 inhibitor (PDB 6LU7) is presented as ribbons. Protomer A, promoter B, and the inhibitor are shown in purple, blue, and yellow, respectively. The His41 and Cys145 residues in the catalytic dyad are shown as yellow spheres. (C) A close-up view of the binding mode of the inhibitor N3 (yellow) in active site of M^{pro}. The key residues are shown in purple sticks, the residues of N-finger are shown in blue sticks. Hydrogen bonds and hydrophobic interactions between the inhibitor N3 and M^{pro} are indicated as green and purple dashed lines, respectively. (D) Cartoon diagram of RdRp representing three subdomains comprising a finger, palm, and thumb, and seven motifs (A-G). (E) Structure of RdRp is presented as ribbons. RdRp has a central cavity composed of three subdomains involved in RNA template and nucleotide binding and catalysis. (F) A close-up view of the central cavity including the RNA template binding site and active site. The RdRp contains seven conserved motifs (motifs A-G) (top). Superposition of elongation complexes from norovirus polymerase (PDB 3H5Y) onto the SARS-CoV-2 RdRp (bottom). RNA template, incoming NTP, and bound catalytic metal ions are shown as orange, cyan, and purple, respectively.

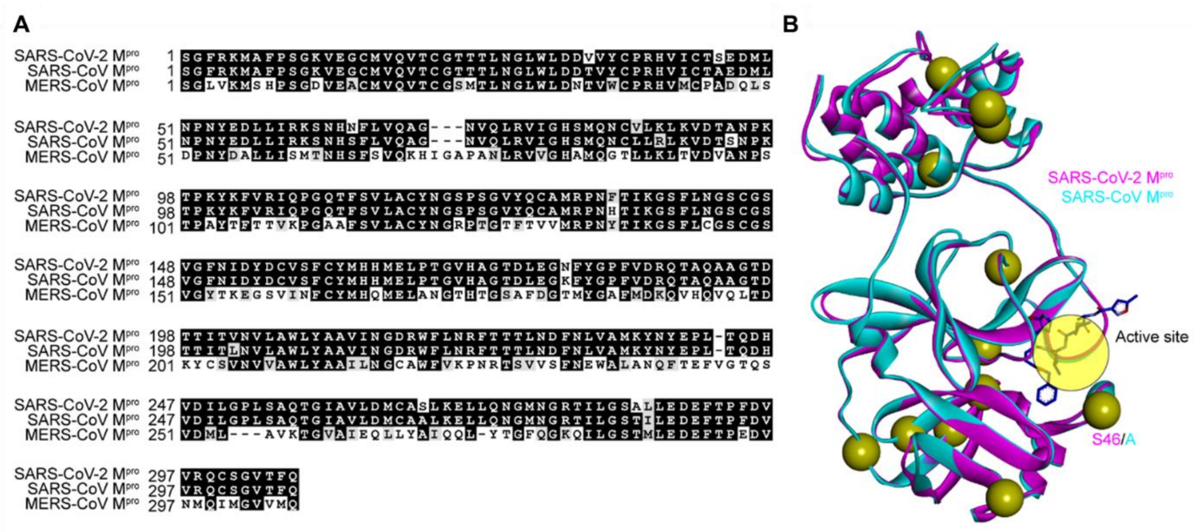

Fig. S3. Sequence and structure alignment of M^{pro}. (A) Sequence alignment of M^{pro} of coronaviruses including SARS-CoV-2, SARS-CoV, and MERS-CoV. (B) Structure alignment of M^{pro} between the SARS-CoV-2 and SARS-CoV. The residues where the mutation occurred are indicated by yellow spheres.

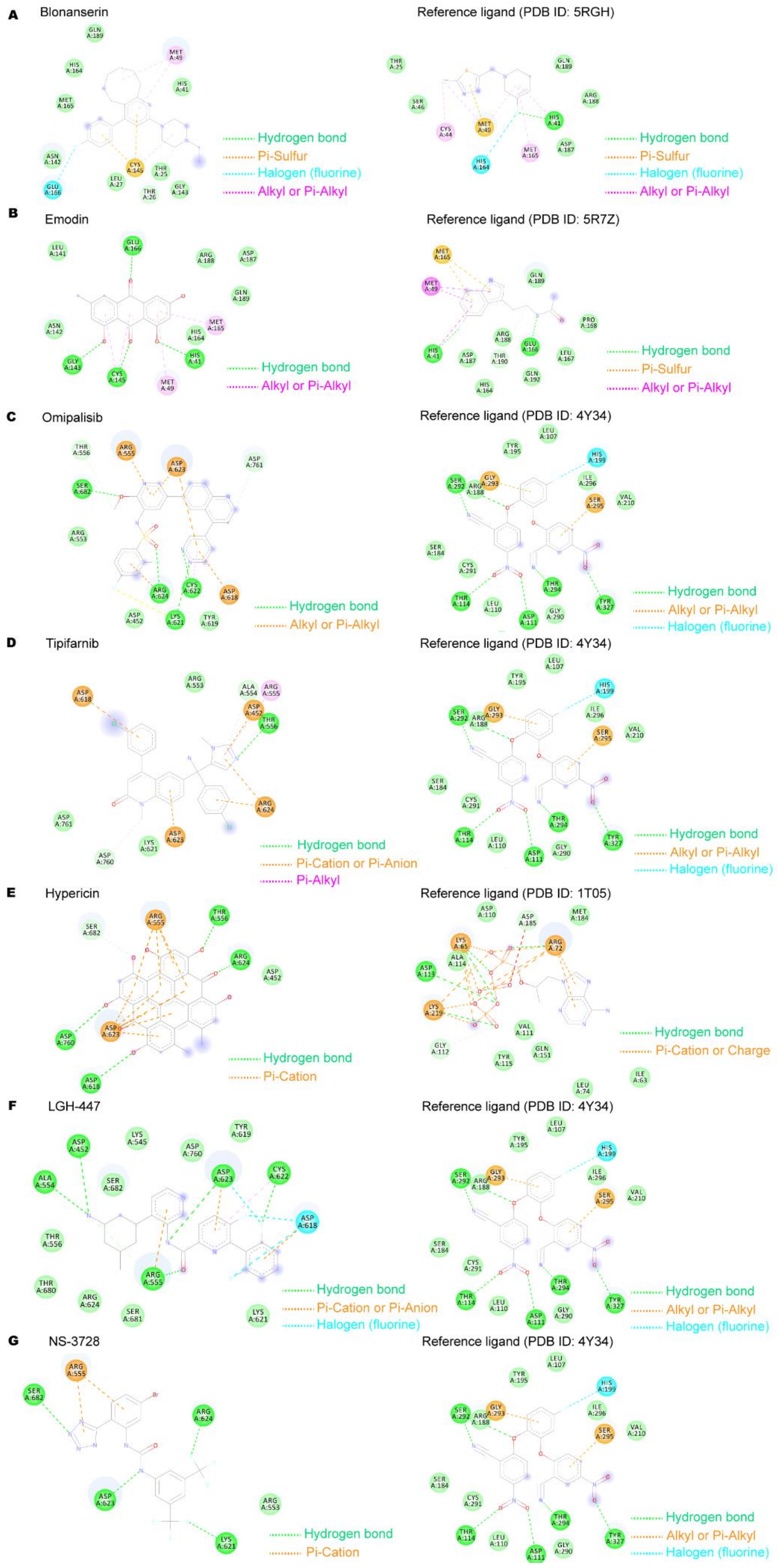
A
Blonanserin
Reference ligand (PDB ID: 5RGH)
Hydrogen bond
Pi-Sulfur
Halogen (fluorine)
Alkyl or Pi-Alkyl
B
Emodin
Reference ligand (PDB ID: 5R7Z)
Hydrogen bond
Alkyl or Pi-Alkyl
Hydrogen bond
Pi-Sulfur
Alkyl or Pi-Alkyl
C
Omipalisib
Reference ligand (PDB ID: 4Y34)
Hydrogen bond
Alkyl or Pi-Alkyl
Hydrogen bond
Alkyl or Pi-Alkyl
Halogen (fluorine)
D
Tipifarnib
Reference ligand (PDB ID: 4Y34)
Hydrogen bond
Pi-Cation or Pi-Anion
Pi-Alkyl
Hydrogen bond
Alkyl or Pi-Alkyl
Halogen (fluorine)
E
Hypericin
Reference ligand (PDB ID: 1T05)
Hydrogen bond
Pi-Cation
Hydrogen bond
Pi-Cation or Charge
F
LGH-447
Reference ligand (PDB ID: 4Y34)
Hydrogen bond
Pi-Cation or Pi-Anion
Halogen (fluorine)
Hydrogen bond
Alkyl or Pi-Alkyl
Halogen (fluorine)
G
NS-3728
Reference ligand (PDB ID: 4Y34)
Hydrogen bond
Pi-Cation
Hydrogen bond
Alkyl or Pi-Alkyl
Halogen (fluorine)

Fig. S4. Interaction profiles between the potent seven drugs with their respective reference compounds in PDB. (A) Binding modes of the blonanserin and the reference compound to M^{pro}. (B) Binding modes of the emodin and the reference compound to M^{pro}. (C) Binding modes of the omipalisib and the reference compound to RdRp. (D) Binding modes of the tipifarnib and the reference compound to RdRp. (E) Binding modes of the hypericin and the reference compound to RdRp. (F) Binding modes of the LGH-447 and the reference compound to RdRp. (G) Binding modes of the NS-3728 and the reference compound to RdRp.

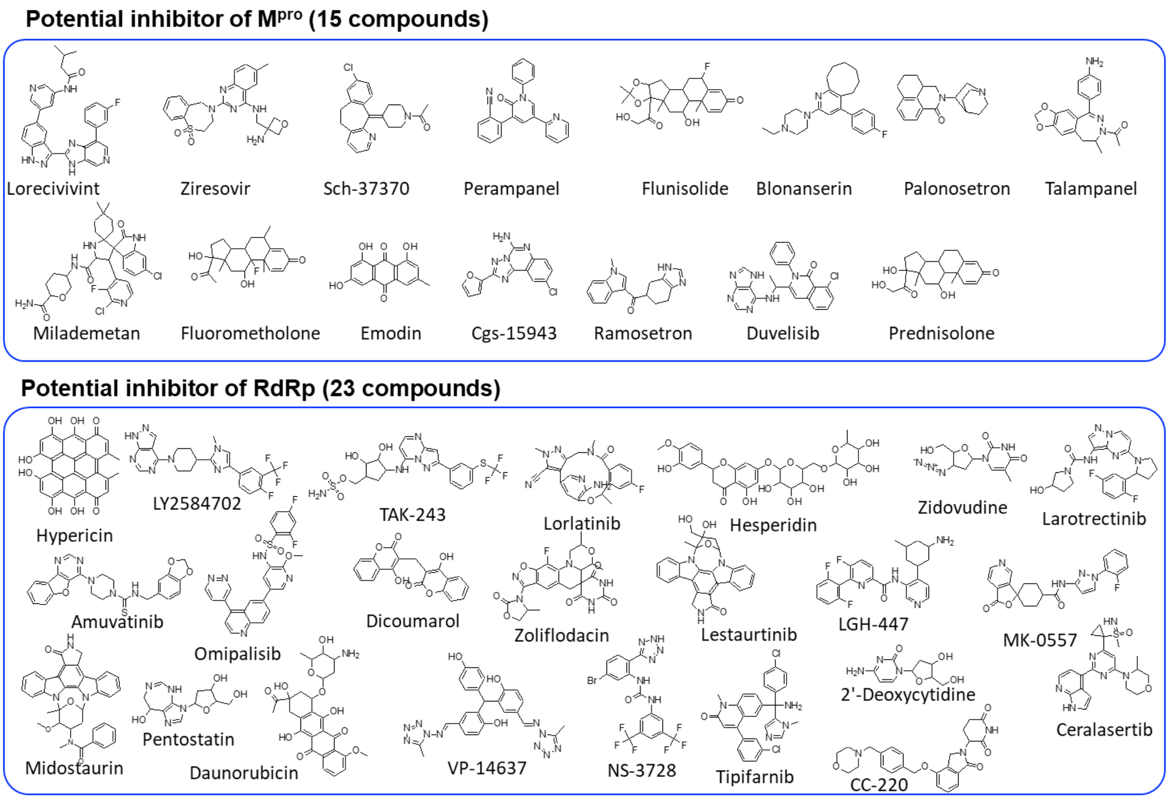

Fig. S5. Potential inhibitors of M^{pro} and RdRp predicted by virtual screening based on molecular docking simulation with pre-docking and post-docking simulations.

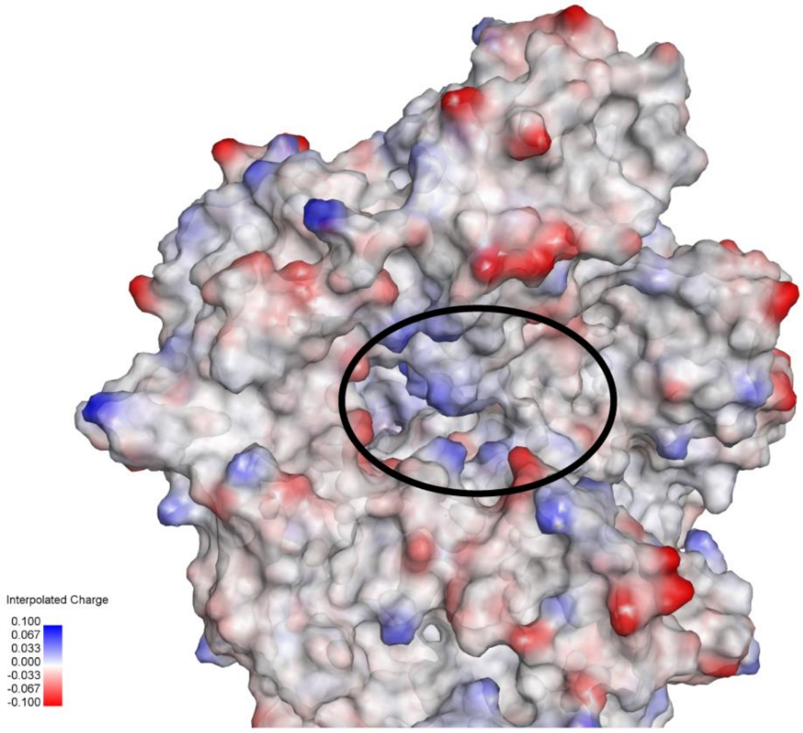

Fig. S6. Surface electrostatics of RdRp. The RNA template and nucleotide binding sites have a positive electrostatic potential as shown in black circle.

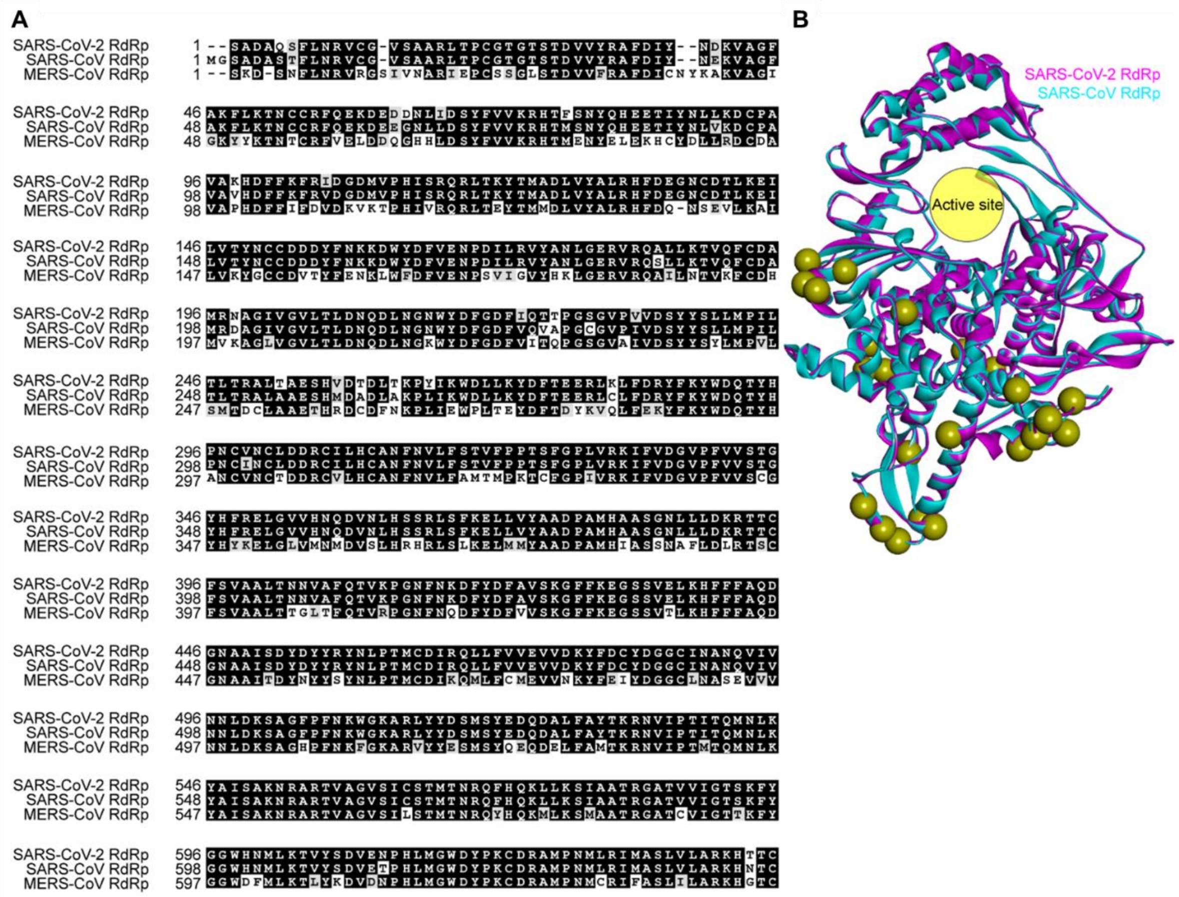

Fig. S7. Sequence and structure alignment of RdRp. (A) Sequence alignment of RdRp of coronaviruses including SARS-CoV-2, SARS-CoV, and MERS-CoV. (B) Structure alignment of RdRp between the SARS-CoV-2 and SARS-CoV. The residues where the mutation occurred are indicated by yellow spheres.

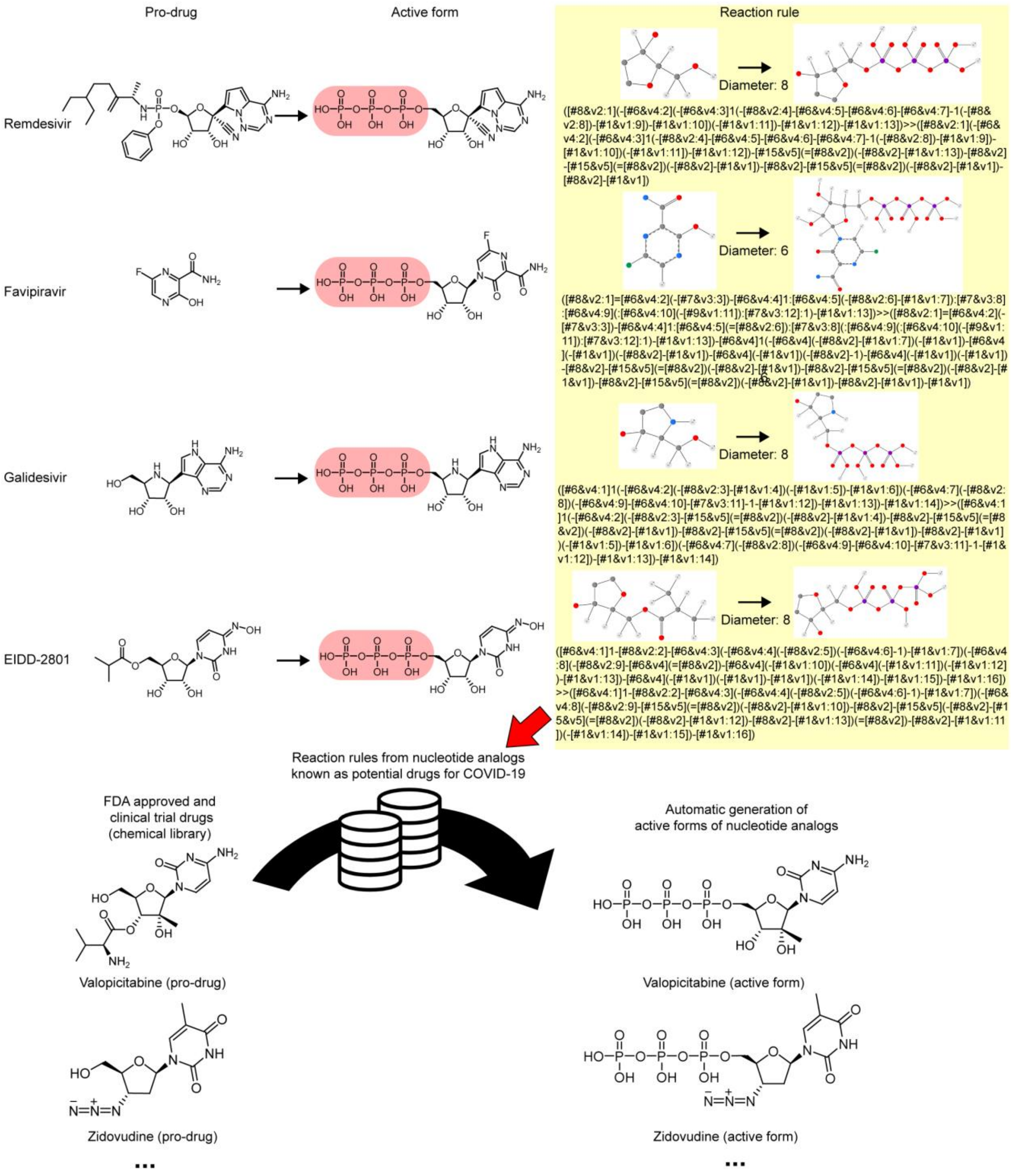

Fig. S8. Automatic conversion of nucleotide analog pro-drugs to their active forms using reaction rules.

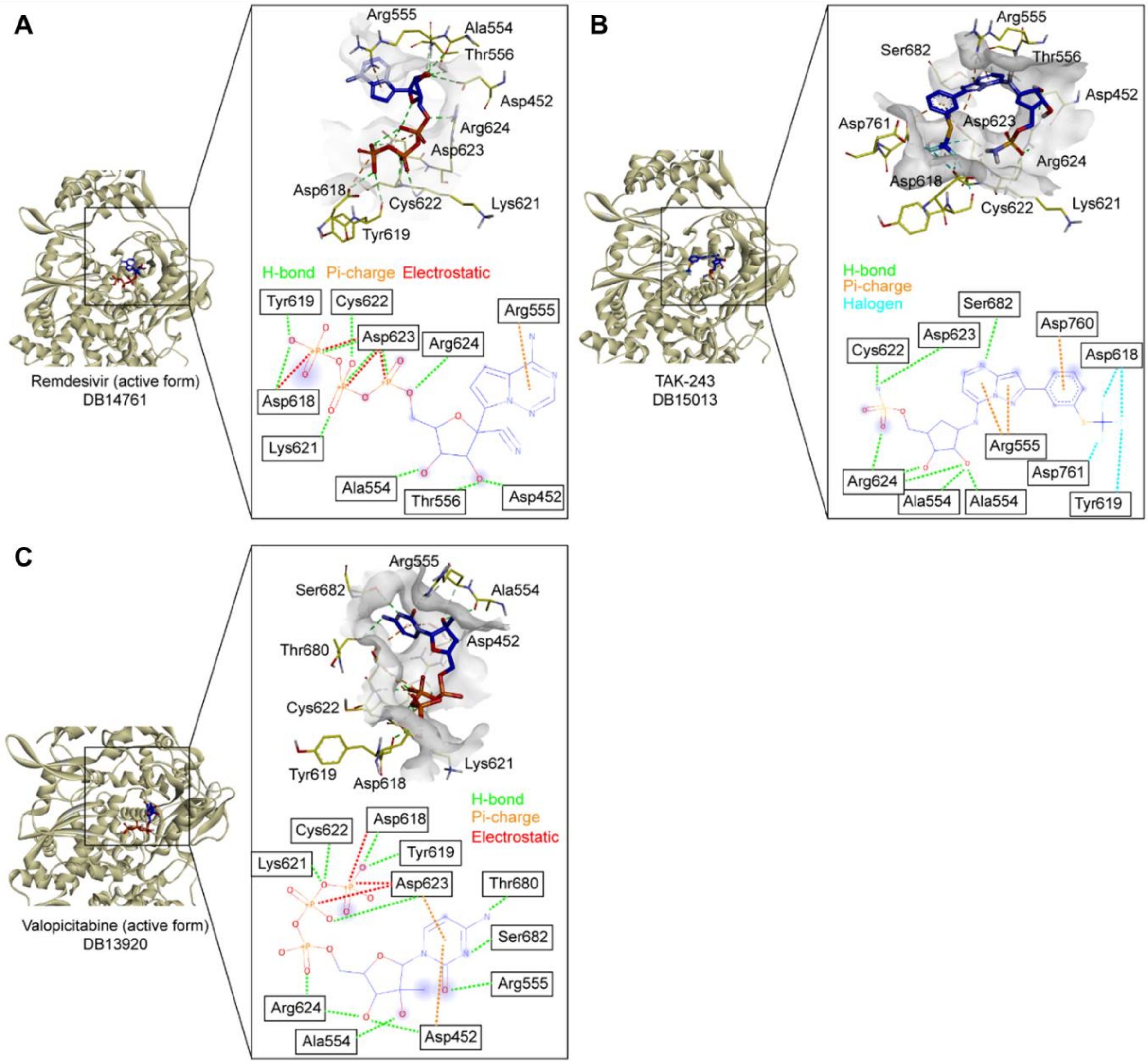

Fig. S9. Molecular docking of nucleotide analog drugs on SARS-CoV-2 RdRp. (A) A binding pose of remdesivir (active form) with RdRp using AutoDock Vina. 3D (top) and 2D (bottom) representations showing the main interactions between the remdesivir and the RdRp are displayed. Hydrogen bond, pi-charge, electrostatic interactions are depicted as green, orange, red dotted lines, respectively. (B) A binding pose of TAK-243 with RdRp using AutoDock Vina. 3D (top) and 2D (bottom) representations showing the main interactions between the TAK-243 and the RdRp are displayed. Hydrogen bond, pi-charge, halogen interactions are depicted as green, orange, cyan dotted lines, respectively. (C) A binding pose of valopicitabine (active form) with RdRp using AutoDock Vina. 3D (top) and 2D (bottom) representations showing the main interactions between the valopicitabine and the RdRp are displayed. Hydrogen bond, pi-charge, electrostatic interactions are depicted as green, orange, red dotted lines, respectively.

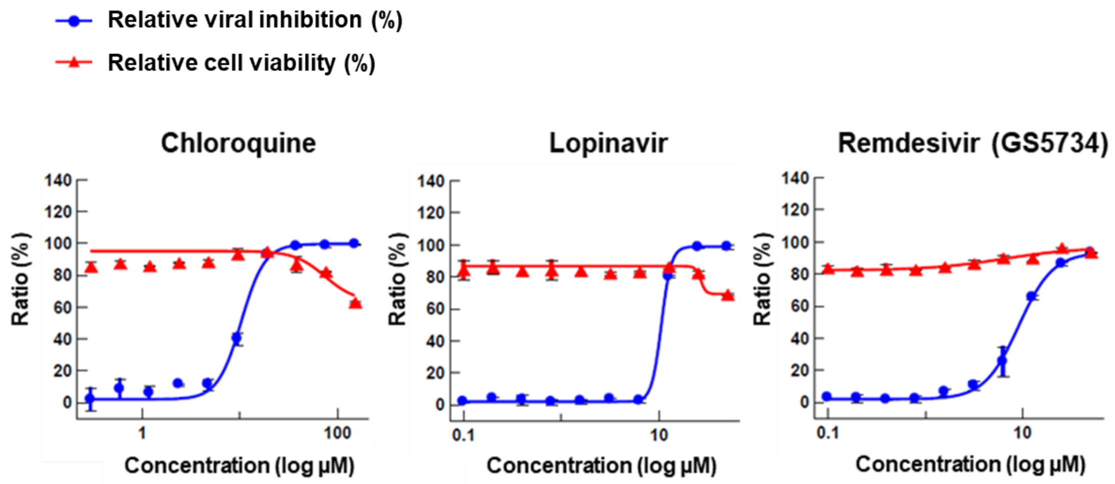

Chloroquine		Lopinavir		Remdesivir	
IC_{50} (μM)	CC_{50} (μM)	IC_{50} (μM)	CC_{50} (μM)	IC_{50} (μM)	CC_{50} (μM)
7.95	>150	11.41	>50	9.49	>50
8.52	>150	10.57	>50	10.22	>50
10.19	>150	11.02	>50	10.55	>50

Fig. S10. Dose-response curves of reference drugs that have previously been identified to inhibit SARS-CoV-2 by immunofluorescence-based assay.

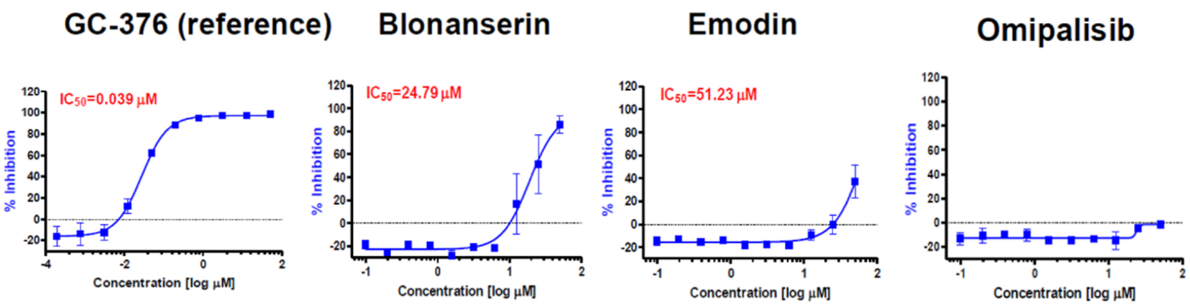

Fig. S11. Inhibitory activity profiles of drug candidates against SARS-CoV-2 M^{pro}. All data are shown as mean ± s.d. of duplicate independent experiments.

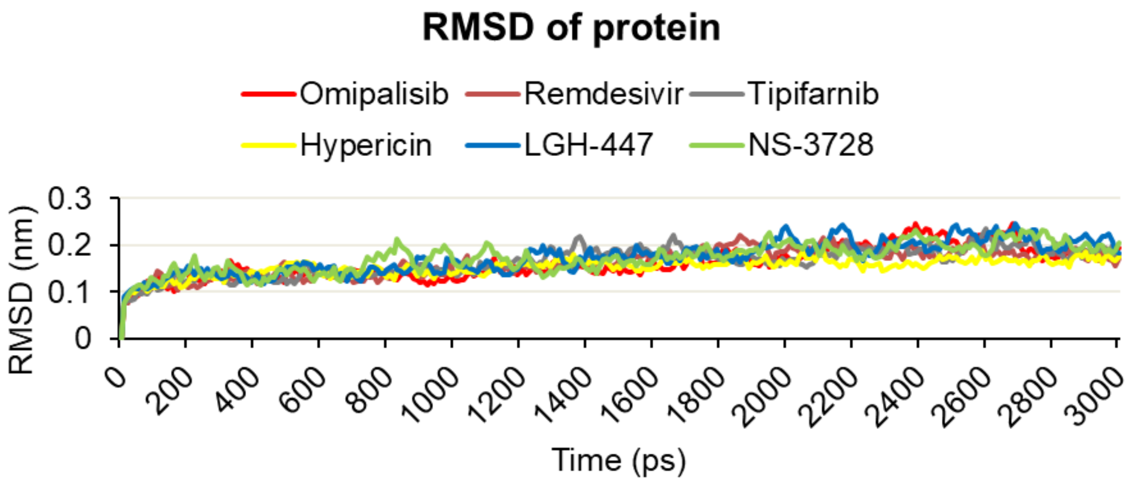

Fig. S12. RMSD of proteins for the six protein-drug complexes during the molecular dynamics simulations. The RMSD values were extracted from protein alpha-carbons of the complex structures, omipalisib (red), remdesivir (brown), tipifarnib (gray), hypericin (yellow), LGH-447 (blue), and NS-3728 (green).

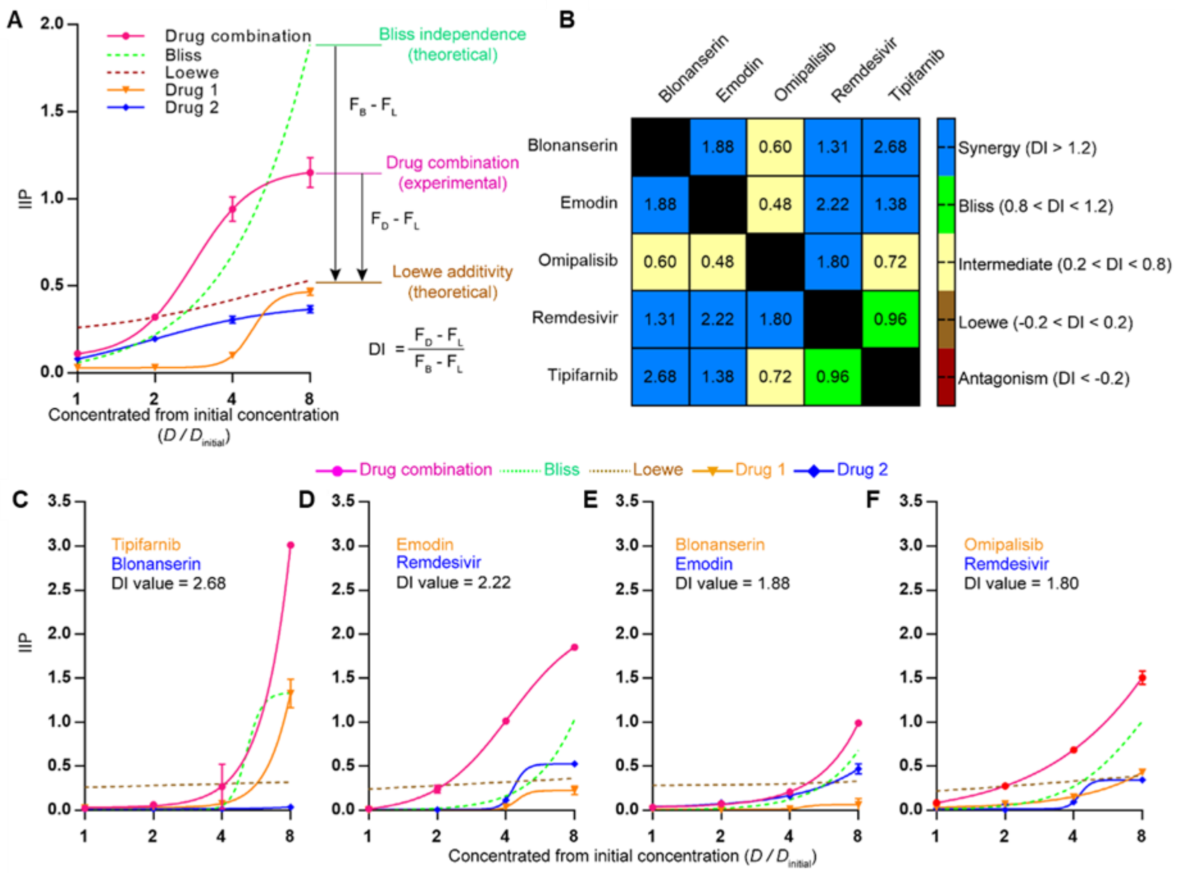

	Blonanserin	Emodin	Omipalisib	Remdesivir	Tipifarnib
Blonanserin		1.88	0.60	1.31	2.68
Emodin	1.88		0.48	2.22	1.38
Omipalisib	0.60	0.48		1.80	0.72
Remdesivir	1.31	2.22	1.80		0.96
Tipifarnib	2.68	1.38	0.72	0.96	

Fig. S13. Instantaneous inhibitory potential (IIP) analyses of drug combinations. (A) IIP plots for drug 1 and drug 2 alone and predictions of the combined effects by the Bliss and Loewe models. DI values were calculated to quantify the combined effects of drug candidates in relation to the Bliss and Loewe models (dashed lines). (B) The observed combination effects categorized by DI values (26): synergy, DI > 1.2; Bliss, 0.8 < DI < 1.2; intermediate, 0.2 < DI < 0.8; Loewe, -0.2 < DI < 0.2, antagonism, DI < -0.2. Quantification of the IIP and DI values of selected drug combinations: (C) tipifarnib/blonanserin, (D) emodin/remdesivir, (E) blonanserin/emodin, and (F) omipalisib/remdesivir.

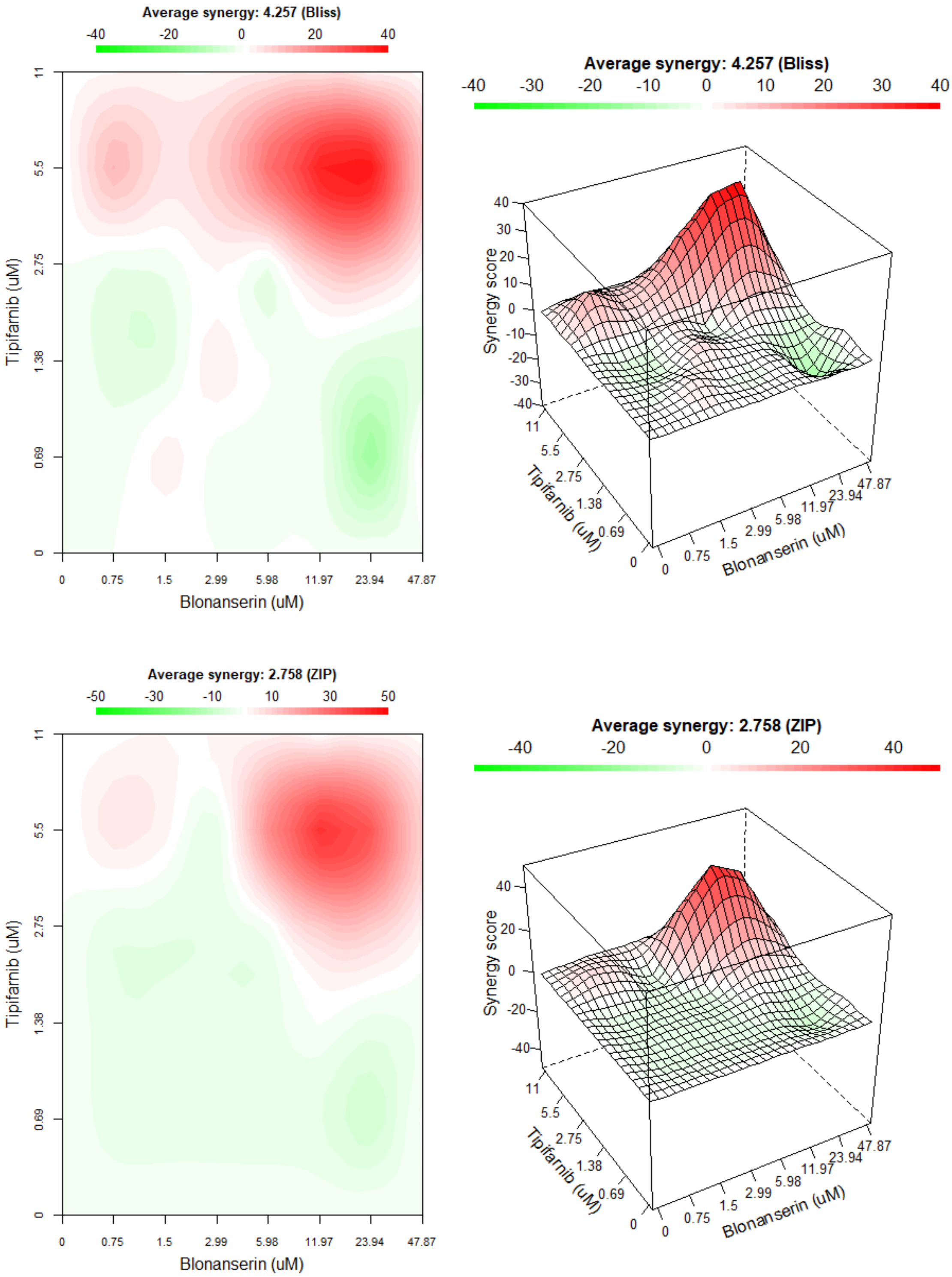

Fig. S14. Synergistic effect of tipifarnib and blonanserin in Vero cells infected with SARS-CoV-2 using the Bliss and ZIP models.

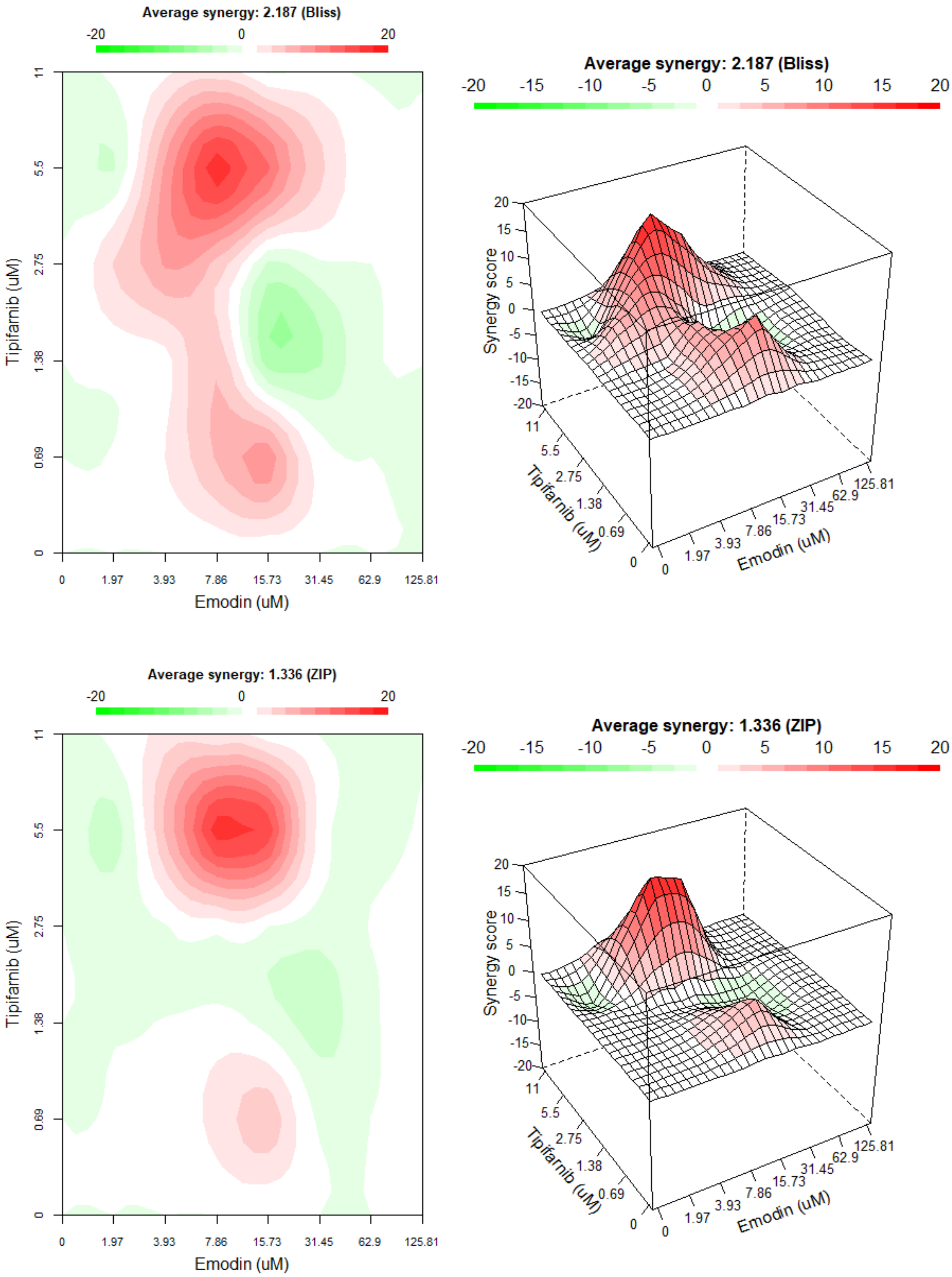

Fig. S15. Synergistic effect of tipifarnib and emodin in Vero cells infected with SARS-CoV-2 using the Bliss and ZIP models.

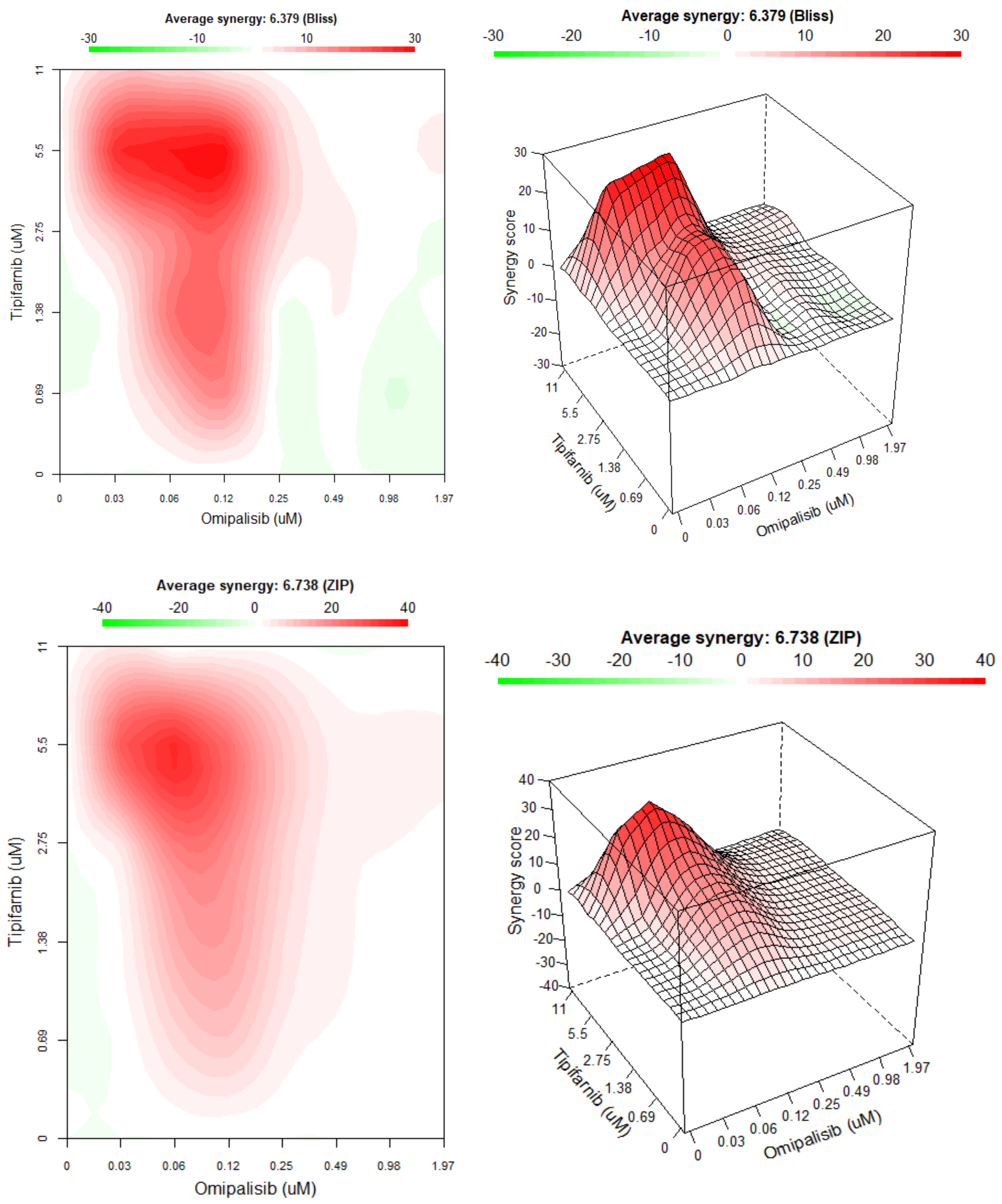

Fig. S16. Synergistic effect of tipifarnib and omipalisib in Vero cells infected with SARS-CoV-2 using the Bliss and ZIP models.

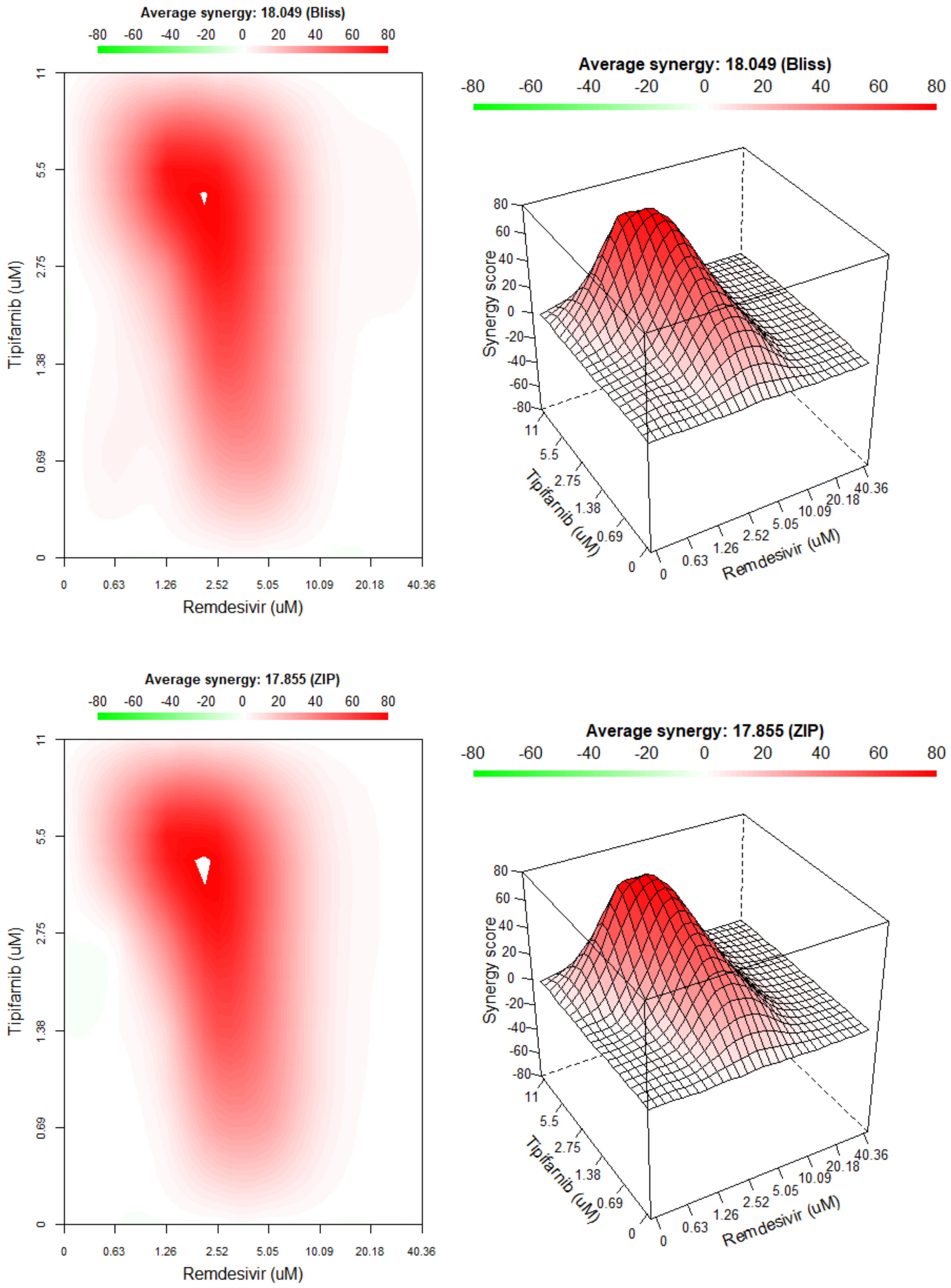

Fig. S17. Synergistic effect of tipifarnib and remdesivir in Vero cells infected with SARS-CoV-2 using the Bliss and ZIP models.

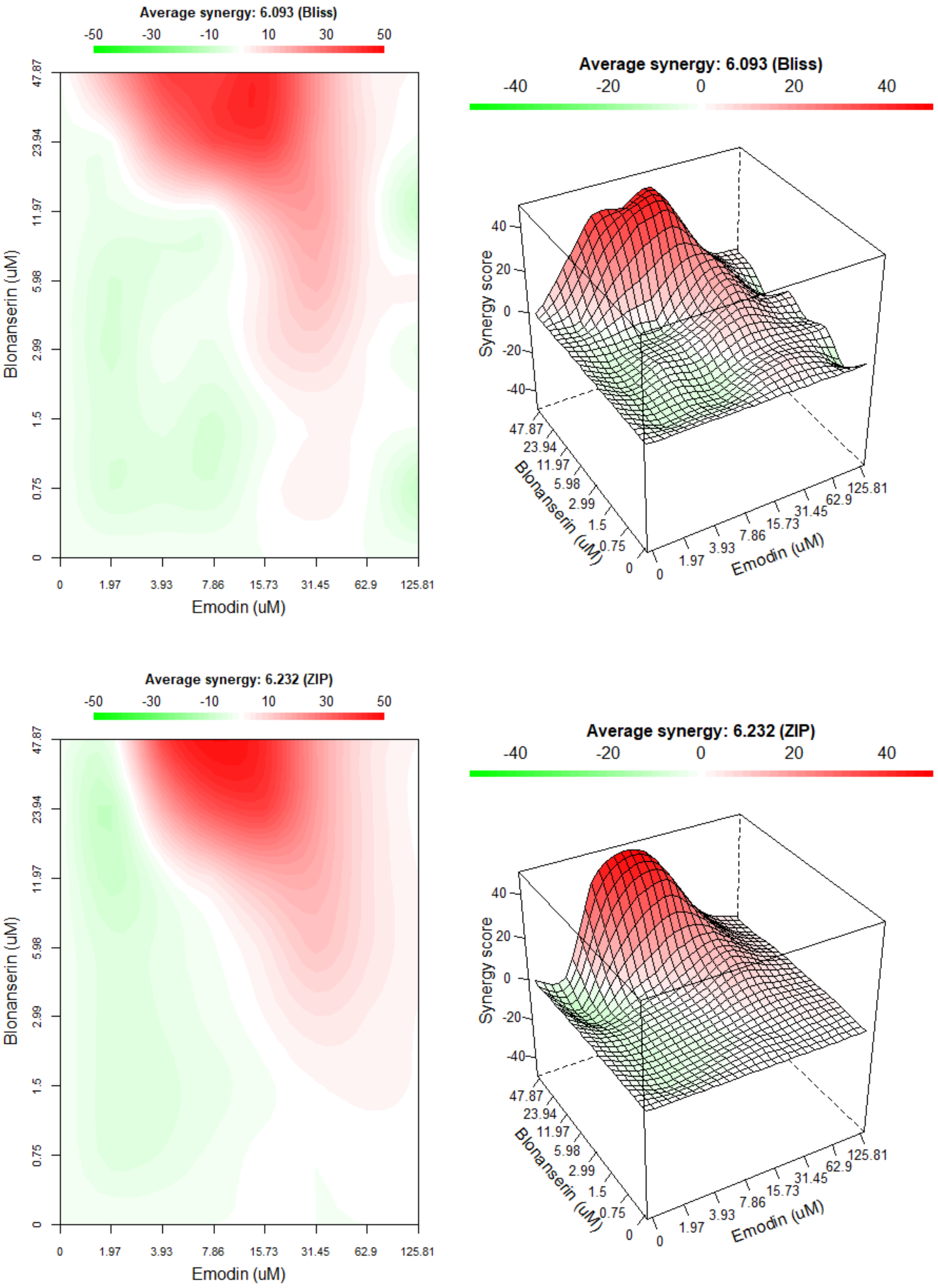

Fig. S18. Synergistic effect of blonanserin and emodin in Vero cells infected with SARS-CoV-2 using the Bliss and ZIP models.

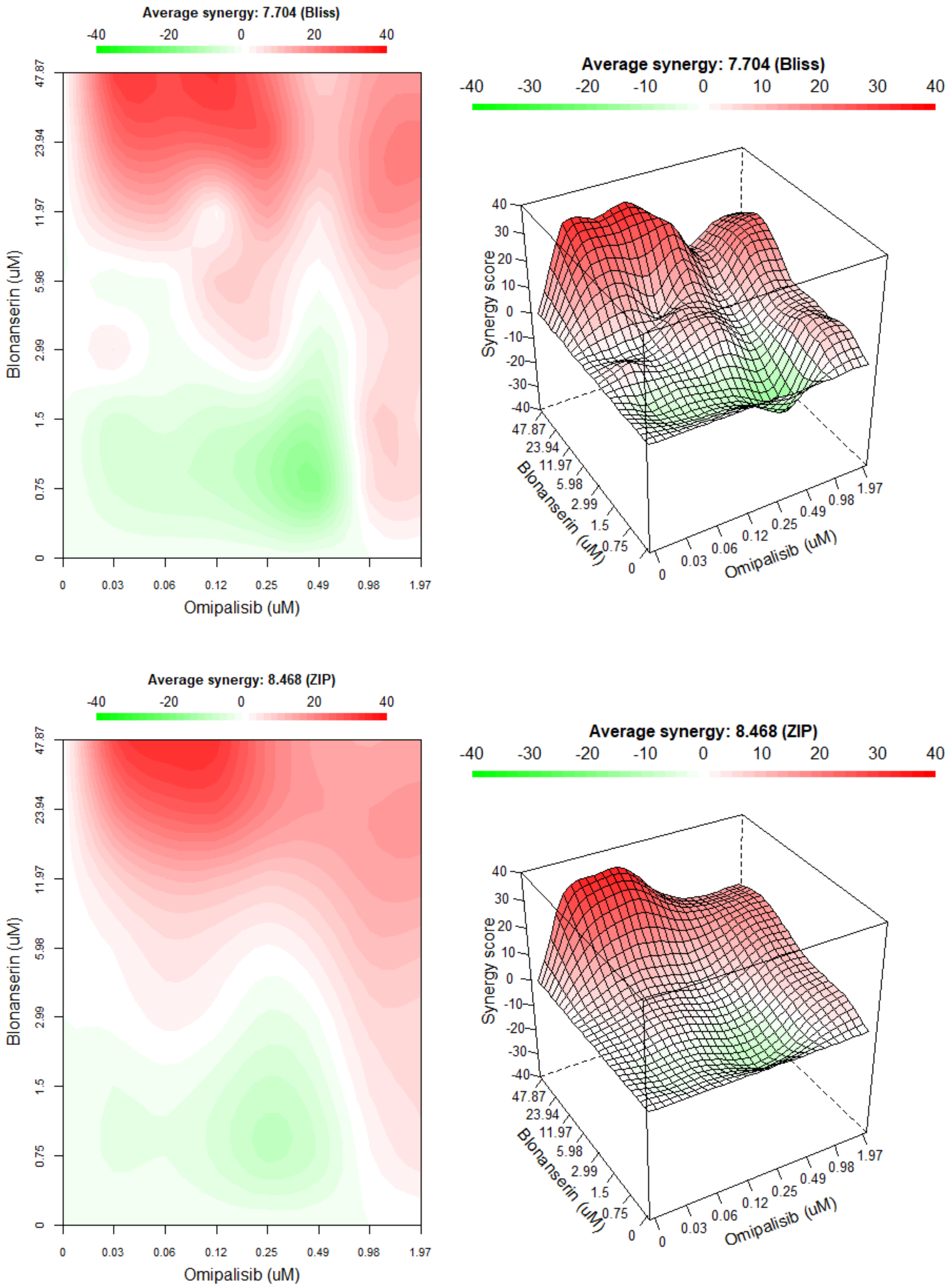

Fig. S19. Synergistic effect of blonanserin and omipalisib in Vero cells infected with SARS-CoV-2 using the Bliss and ZIP models.

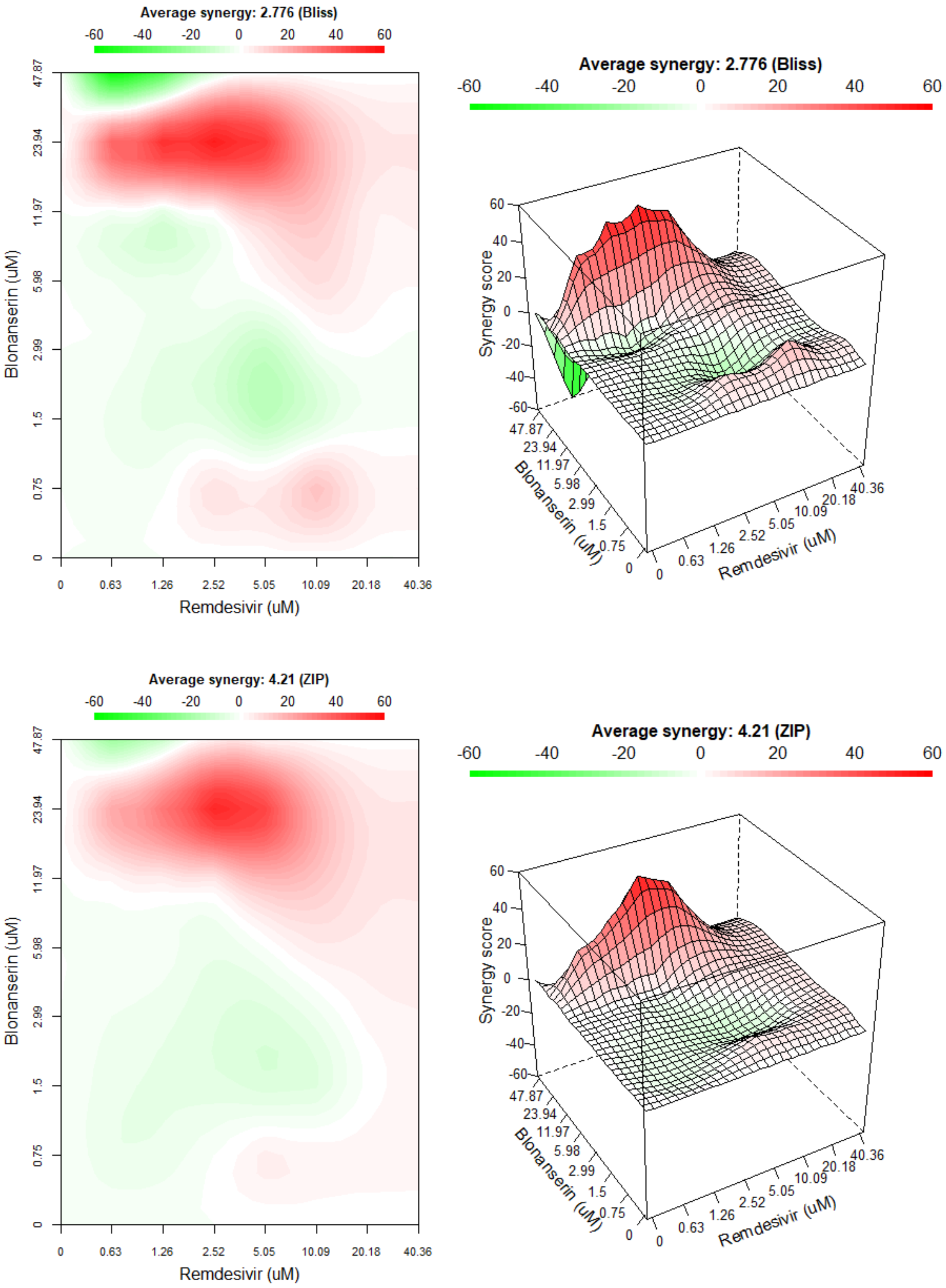

Fig. S20. Synergistic effect of blonanserin and remdesivir in Vero cells infected with SARS-CoV-2 using the Bliss and ZIP models.

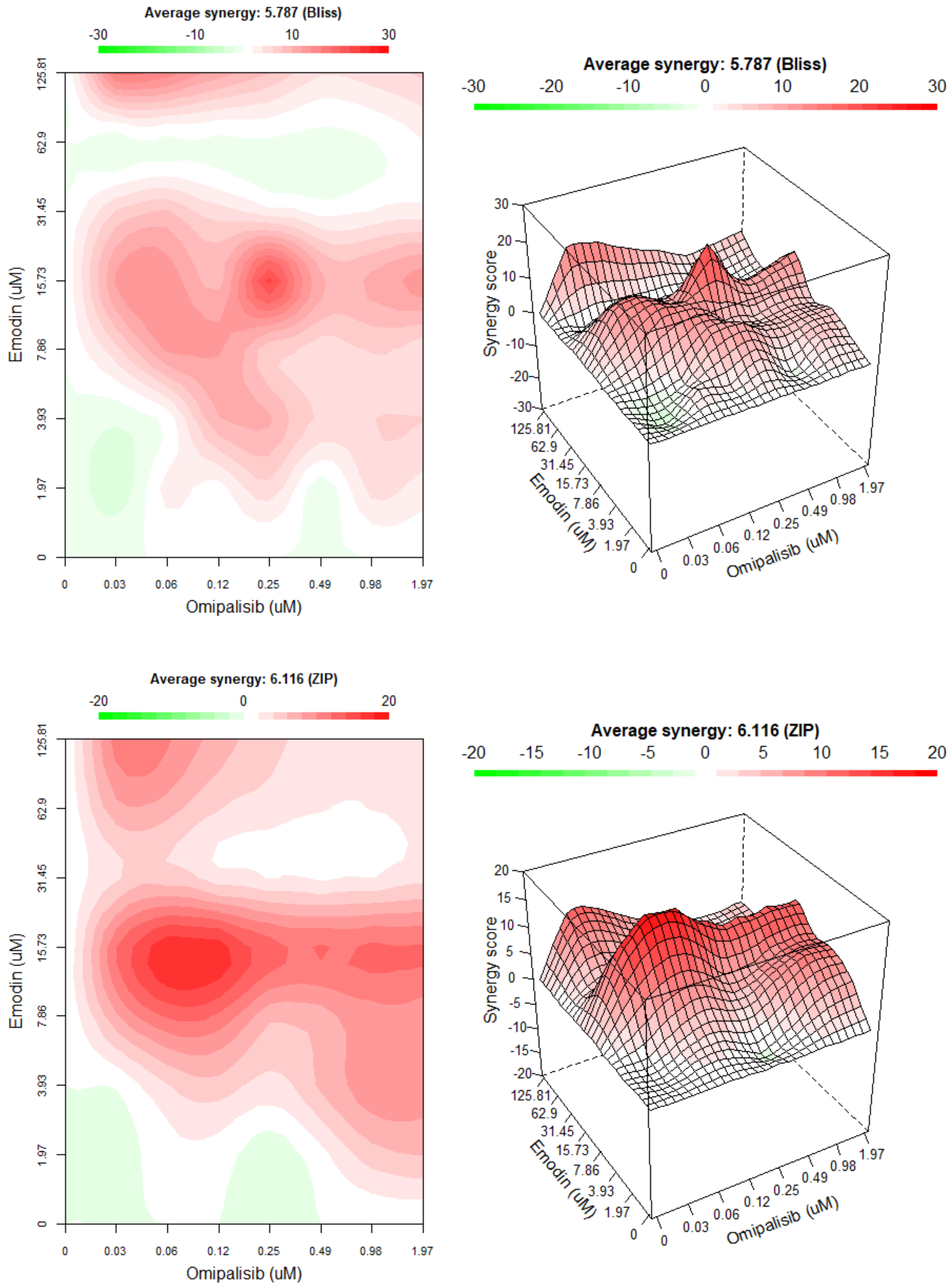

Fig. S21. Synergistic effect of emodin and omipalisib in Vero cells infected with SARS-CoV-2 using the Bliss and ZIP models.

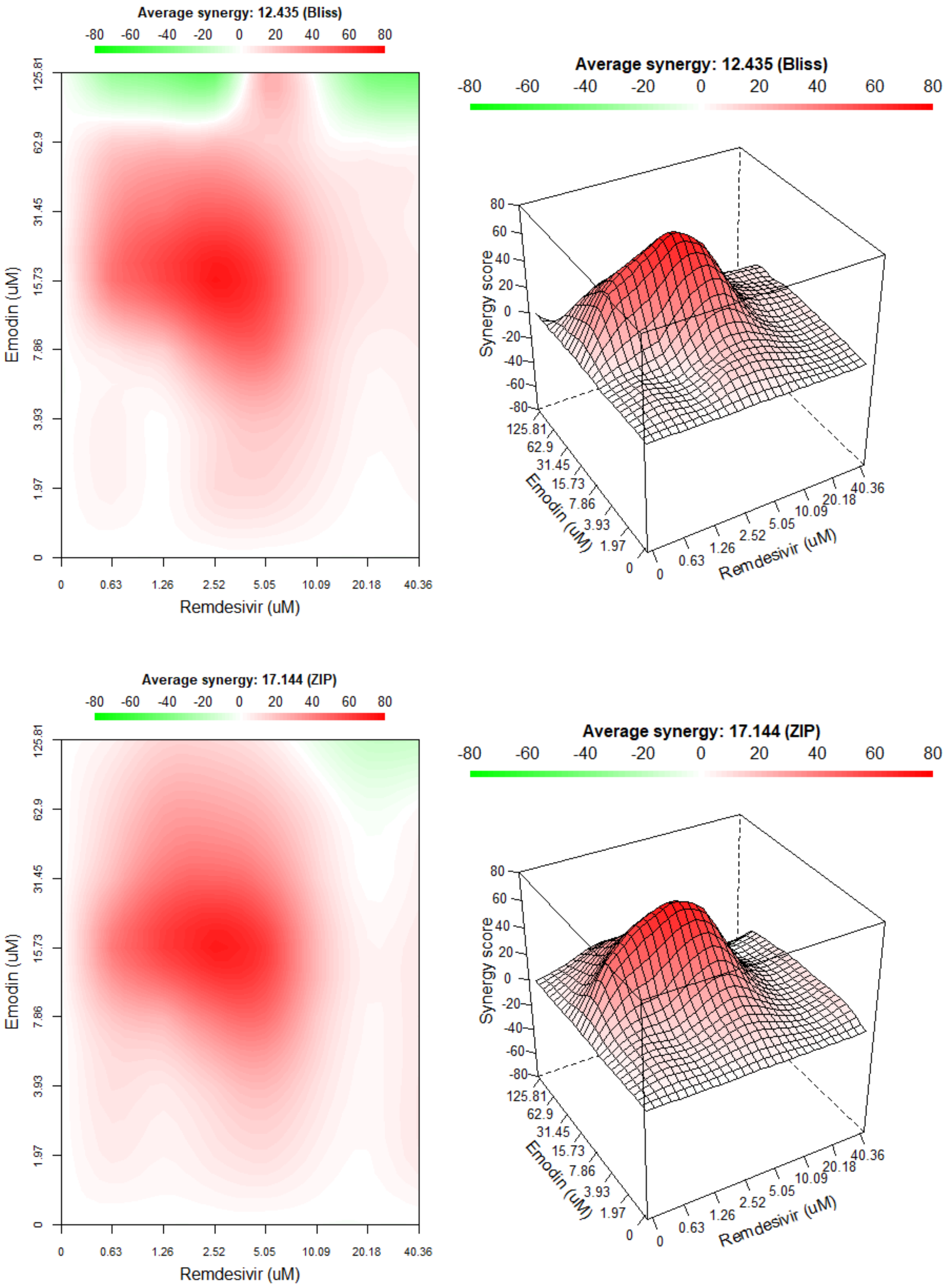

Fig. S22. Synergistic effect of emodin and remdesivir in Vero cells infected with SARS-CoV-2 using the Bliss and ZIP models.

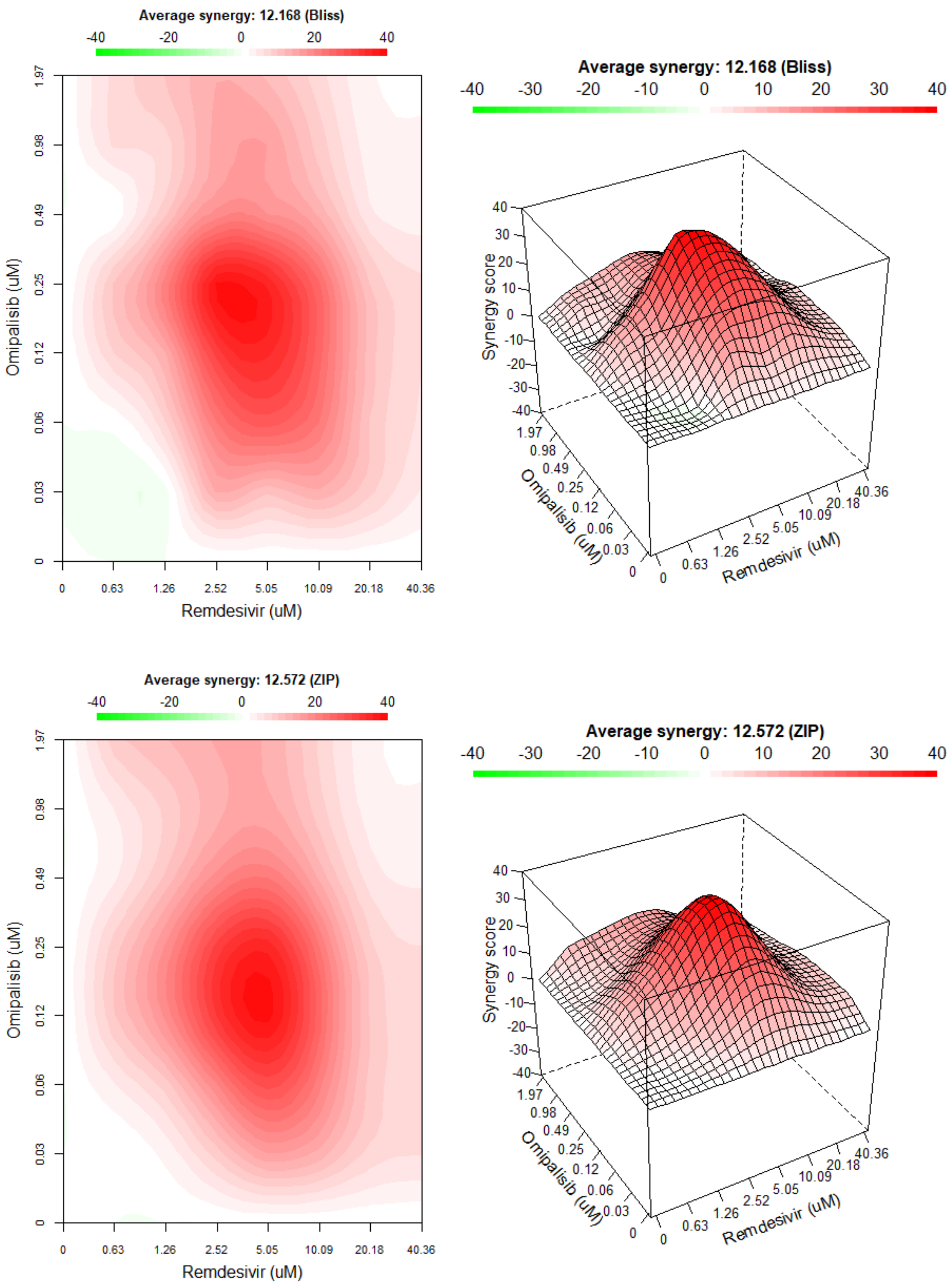

Fig. S23. Synergistic effect of omipalisib and remdesivir in Vero cells infected with SARS-CoV-2 using the Bliss and ZIP models.

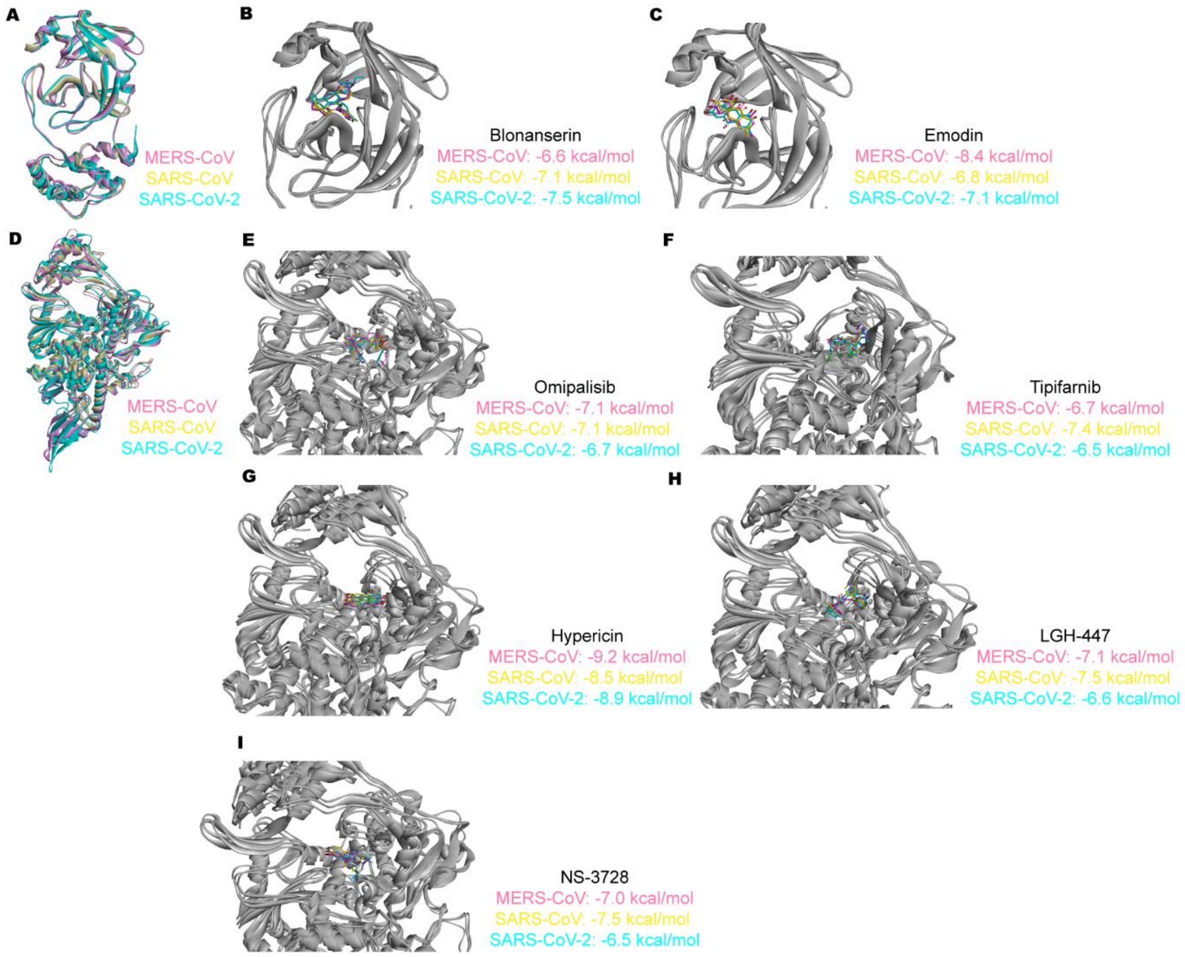

Fig. S24. Molecular docking of drug candidates on M^{pro} and RdRp of coronaviruses using AutoDock Vina. (A) Structure alignment of M^{pro} between the SARS-CoV-2, SARS-CoV, and MERS-CoV. (B) Binding mode of blonanserin to M^{pro} of coronaviruses. (C) Binding mode of emodin to M^{pro} of coronaviruses. (D) Structure alignment of RdRp between the SARS-CoV-2, SARS-CoV, and MERS-CoV. (E) Binding mode of omipalisib to RdRp of coronaviruses. (F) Binding mode of tipifarnib to RdRp of coronaviruses. (G) Binding mode of hypericin to RdRp of coronaviruses. (H) Binding mode of LGH-447 to RdRp of coronaviruses. (I) Binding mode of NS-3728 to RdRp of coronaviruses.

Table S1. Twenty-five active ligands for M^{pro} from the co-crystal structures in PDB.

PDB ID	Ligand	Organism	Binding affinity to M^{pro} (IC_{50}, µM)
**Peptidomimetic inhibitor**			
6LU7	N3	SARS-CoV-2	N/A
6Y2F	O6K	SARS-CoV-2	0.67 (1)
**Non-peptidomimetic inhibitor**			
6W63	X77	SARS-CoV-2	2.3 (27)
**Fragment (lead-like)**			
5R7Y	JFM	SARS-CoV-2	N/A
5R7Z	HWH	SARS-CoV-2	N/A
5RE4	SZY	SARS-CoV-2	N/A
5R80	RZG	SARS-CoV-2	N/A
5R81	RZJ	SARS-CoV-2	N/A
5R82	RZS	SARS-CoV-2	N/A
5RE9	LPZ	SARS-CoV-2	N/A
5REB	T0Y	SARS-CoV-2	N/A
5RGH	U0M	SARS-CoV-2	N/A
5RGI	U0P	SARS-CoV-2	N/A
5RGK	U0V	SARS-CoV-2	N/A
5R83	K0G	SARS-CoV-2	N/A
5REH	AWP	SARS-CoV-2	N/A
5R84	GWS	SARS-CoV-2	N/A
5REZ	T54	SARS-CoV-2	N/A
5RF1	T5G	SARS-CoV-2	N/A
5RG1	T9J	SARS-CoV-2	N/A
5RF2	HVB	SARS-CoV-2	N/A
5RF3	T5V	SARS-CoV-2	N/A
5RF6	NTG	SARS-CoV-2	N/A
5RF7	T67	SARS-CoV-2	N/A
5RFE	JGG	SARS-CoV-2	N/A

Table S2. Eight active ligands for RdRp from the co-crystal structures in PDB.

PDB ID	Ligand	Organism	Binding affinity to RdRp (IC_{50}, μM)
**Nucleotide analog**			
7BV2	F86 (remdesivir)	SARS-CoV-2	N/A
4WTG	6GS (sofosbuvir)	Hepatitis C virus	0.064 (28)
1T05	TNV (tenofovir)	HIV-1	1.3 (29)
3N6M	GTP	Enterovirus A71	N/A
2F8E	U5P	Foot-and-mouth disease virus	N/A
3H5Y	CTP	Norwalk virus	N/A
**Non-nucleotide analog**			
4LQ9	21D	Norovirus	14.0 (30)
4Y34	45Z	Coxsackievirus B3	N/A

Table S3. Hit compounds with anti-SARS-CoV-2 activity in Vero and Calu-3 cells.

Name (DrugBank ID)	Clinical phase*	Indication†	Docking energy‡ (kcal/mol)	Approx. C_{max}§	SARS-CoV-2 IC_{50} in Vero cells (μM)	CC_{50} in Vero cells (μM)	SARS-CoV-2 IC_{50} in Calu-3 cells (μM)	CC_{50} in Calu-3 cells (μM)
**M^{pro} target**								
Blonanserin (DB09223)	Approved	Schizophrenia	-7.5	3 nM (human, oral) (31)	11.97	30.30	> 50	> 50
Emodin (DB07715)	Phase 1	Polycystic kidney	-7.1	78 nM (rat, oral) (32)	31.45	> 50	27.87	> 50
**RdRp target**								
Omipalisib (DB12703)	Phase 1	Idiopathic pulmonary fibrosis and solid tumors	-6.7	0.3 μM (human, oral) (33, 34)	0.49	30.79	0.01	> 0.02
Remdesivir (DB14761)	FDA approved	Ebola virus infection and COVID-19	-7.1	9.0 μM (human, intravenous injection) (35)	10.09	> 50	2.49	> 50
Tipifarnib (DB04960)	Phase 3	Colorectal cancer, leukemia, pancreatic cancer, and solid tumors	-6.5	8.6 μM (human, oral) (36)	11.01	16.57	4.57	> 50
Hypericin (DB13014)	Phase 3	Cutaneous T-cell lymphoma and human immunodeficiency virus Infection	-8.9	8 nM (human, oral) (37)	19.34	> 50	> 50	> 50
LGH-447 (DB14943)	Phase 1	Relapsed and refractory multiple myeloma	-6.6	9.0 μM (mouse, oral) (38)	23.91	> 50	> 50	30.70
NS-3728 (DB05835)	Phase 1	Anemia and cancers	-6.5	36.5 μM (human, oral) (39)	28.37	> 50	> 50	> 50

* Maximum clinical phase information of each drug was referenced on the DrugBank (https://go.drugbank.com/), if the drug was not available in DrugBank, the drug information was retrieved from related literature or patents.
† Indication of each drug was referenced on the DrugBank (https://go.drugbank.com/), if the drug was not available in DrugBank, the drug information was retrieved from related literature or patents.
‡ Docking energy was obtained using AutoDock Vina.
§ C_{max} is the maximum serum concentration.

Table S4. The calculated binding energies of six drugs targeting RdRp.

Energy*	Remdesivir	Omipalisib	Tipifarnib	Hypericin	LGH-447	NS-3728
ΔE_{vdw}	-144.129 ± 13.182	-139.318 ± 6.378	-119.243 ± 7.615	-216.876 ± 12.939	-147.003 ± 23.018	-105.825 ± 12.986
ΔE_{ele}	-642.649 ± 96.017	-273.629 ± 27.725	-191.495 ± 56.307	-140.313 ± 50.740	-239.627 ± 44.749	-211.447 ± 39.161
ΔG_{pb}	575.610 ± 61.161	219.503 ± 20.948	127.416 ± 39.894	176.482 ± 32.451	223.821 ± 51.340	128.621 ± 35.655
ΔG_{np}	-19.975 ± 1.046	-14.541 ± 1.149	-17.645 ± 1.164	-15.963 ± 0.849	-17.425 ± 2.506	-10.378 ± 1.835
ΔG_{cal}	-231.142 ± 45.234	-207.985 ± 8.991	-200.967 ± 34.884	-196.670 ± 19.943	-180.234 ± 20.149	-199.029 ± 25.393

* ΔE_{vdw}, van der Waals energy terms; ΔE_{ele}, electrostatic energy; ΔG_{pb}, polar solvation free energy; ΔG_{np}, nonpolar solvation free energy; ΔG_{cal}, final estimated binding free energy (kJ/mol).

Note S1. Instantaneous inhibitory potential (IIP) from the dose-response curves of the antiviral drugs.

The dose-response curve of a single antiviral drug can be analyzed based on the median-effect equation (equation (1)) (40):

$$f_u = \frac{1}{1+\left(\frac{D}{IC_{50}}\right)^m} \qquad (1)$$

Here, f_u is the fraction of infection events unaffected by the drug (i.e., $1 - f_u$ equals the fraction of drug-affected events). *D* is the drug concentration, IC_{50} is the 50% antiviral concentration, and *m* is the dose-response curve slope. Dose-response curves for compounds with higher *m* values show higher antiviral activity at the same normalized concentration so long as the concentration is higher than IC_{50}.

The antiviral activities of compounds can be expressed as the IIP (equation (2)) (26, 41, 42):

$$\mathrm{IIP} = \log\left(\frac{1}{f_u}\right) = \log\left[1 + \left(\frac{D}{IC_{50}}\right)^m\right] \qquad (2)$$

Here, *fu* is the fraction of infection events unaffected by the drug, *D* is the drug concentration, IC_{50} is the 50% antiviral concentration, and *m* is the dose-response curve slope. If a drug reduces SARS-CoV-2 replication by 1 log then *fu* = 0.1 and its IIP = 1, whereas if it reduces viral replication by 2 logs, i.e. 100-fold, its IIP = 2. Importantly, IIP focuses on the remarkable effect of the slope parameter on antiviral activity.

Note S2. Drug combinations with synergistic antiviral activity assessed by the DI values.

Drug combinations can be characterized by two fundamental indices, the Loewe additivity and Bliss independence. We evaluated the drug combinations for Loewe additivity and Bliss independence, because there have been successful cases using these two fundamental indices to evaluate the combined effects of antiviral drugs for HIV-1 and HCV (26, 42). The Loewe additivity is based on isobolograms and assumes similar mechanism or competition for the same binding site. For positive inhibitory slopes, Loewe additivity is described by equation (3):

$$1 = \frac{D_1}{IC_{50_1}\left(\frac{f_{u1+2}}{1-f_{u1+2}}\right)^{-1/m1}} + \frac{D_2}{IC_{50_2}\left(\frac{f_{u1+2}}{1-f_{u1+2}}\right)^{-1/m2}} \qquad (3)$$

Equation (3) is numerically solved for f_{u1+2} to predict the additive effects of the drug combinations.

Bliss independence assumes that each drug acts on different target, and is defined as equation (4):

$$f_{u1+2} = f_{u1} \times f_{u2} = \frac{1}{1+\left(\frac{D_1}{IC_{50_1}}\right)^{m_1}} \times \frac{1}{1+\left(\frac{D_2}{IC_{50_2}}\right)^{m_2}} \qquad (4)$$

where f_{u1+2}, f_{u1} and f_{u2} are the fractions of infection events unaffected by the combined drugs A and B, drug A, and drug B, respectively. Using equation (4), we determined the antiviral effects of combined drugs A and B, $1 - f_{u1+2}$, from the antiviral effects of each single drugs.

To quantify the independence of each drug, Jilek et al. (26) proposed a new index called the degree of independence (DI):

$$\mathrm{DI} = \frac{F_E - F_L}{F_B - F_L} \qquad (5)$$

where F_E, F_B and F_L denote the logarithmic drug effects ($\log[(1 - f_u^{u1+2})/f_u^{u1+2}]$) of experimental data, Bliss independence and Loewe additivity, respectively. Note that this index incorporates both Bliss independence and Loewe additivity, and categorizes the experimental data of combination effects. From the DI values calculated by equation (5), the anvi-SARS-CoV-2 effects of drug combinations can be assessed (*SI Appendix*, Fig. S10).

SI References

1. L. Zhang *et al.*, Crystal structure of SARS-CoV-2 main protease provides a basis for design of improved alpha-ketoamide inhibitors. *Science* **368**, 409-412 (2020).
2. Z. Jin *et al.*, Structure of M(pro) from COVID-19 virus and discovery of its inhibitors. *Nature* **582**, 289-293 (2020).
3. S. Pronk *et al.*, GROMACS 4.5: a high-throughput and highly parallel open source molecular simulation toolkit. *Bioinformatics* **29**, 845-854 (2013).
4. Y. Gao *et al.*, Structure of the RNA-dependent RNA polymerase from COVID-19 virus. *Science* **368**, 779-782 (2020).
5. A. D. MacKerell, Jr., N. Banavali, N. Foloppe, Development and current status of the CHARMM force field for nucleic acids. *Biopolymers* **56**, 257-265 (2000).
6. W. L. Jorgensen, J. Chandrasekhar, J. D. Madura, R. W. Impey, M. L. Klein, Comparison of simple potential functions for simulating liquid water. *J. Chem. Phys.* **79**, 926-935 (1983).
7. T. Darden, D. York, L. Pedersen, Particle mesh Ewald: An *N*· log (*N*) method for Ewald sums in large systems. *J Chem. Phys.* **98**, 10089-10092 (1993).
8. D. F. Zamyatkin, F. Parra, A. Machin, P. Grochulski, K. K. Ng, Binding of 2'-amino-2'-deoxycytidine-5'-triphosphate to norovirus polymerase induces rearrangement of the active site. *J. Mol. Biol.* **390**, 10-16 (2009).
9. W. Yin *et al.*, Structural basis for inhibition of the RNA-dependent RNA polymerase from SARS-CoV-2 by remdesivir. *Science* **368**, 1499-1504 (2020).
10. D. S. Wishart *et al.*, DrugBank 5.0: a major update to the DrugBank database for 2018. *Nucleic Acids Res.* **46**, D1074-D1082 (2018).
11. T. Sterling, J. J. Irwin, ZINC 15-ligand discovery for everyone. *J. Chem. Inf. Model.* **55**, 2324-2337 (2015).
12. A. Gaulton *et al.*, The ChEMBL database in 2017. *Nucleic Acids Res.* **45**, D945-D954 (2017).
13. G. Landrum (2006) RDKit: Open-source cheminformatics (http://www.rdkit.org).
14. T. Duigou, M. du Lac, P. Carbonell, J. L. Faulon, RetroRules: a database of reaction rules for engineering biology. *Nucleic Acids Res.* **47**, D1229-D1235 (2019).
15. S. Riniker, G. A. Landrum, Better informed distance geometry: using what we know to improve conformation generation. *J. Chem. Inf. Model.* **55**, 2562-2574 (2015).
16. P. J. Ballester, W. G. Richards, Ultrafast shape recognition to search compound databases for similar molecular shapes. *J. Comput. Chem.* **28**, 1711-1723 (2007).
17. A. M. Schreyer, T. Blundell, USRCAT: real-time ultrafast shape recognition with pharmacophoric constraints. *J. Cheminform.* **4**, 27 (2012).
18. M. S. Armstrong *et al.*, ElectroShape: fast molecular similarity calculations incorporating shape, chirality and electrostatics. *J. Comput. Aided Mol. Des.* **24**, 789-801 (2010).
19. M. Wojcikowski, P. Zielenkiewicz, P. Siedlecki, Open Drug Discovery Toolkit (ODDT): a new open-source player in the drug discovery field. *J. Cheminform.* **7**, 26 (2015).
20. O. Trott, A. J. Olson, AutoDock Vina: improving the speed and accuracy of docking with a new scoring function, efficient optimization, and multithreading. *J. Comput. Chem.* **31**, 455-461 (2010).
21. N. M. O'Boyle *et al.*, Open Babel: An open chemical toolbox. *J. Cheminform.* **3**, 33 (2011).
22. S. Salentin, S. Schreiber, V. J. Haupt, M. F. Adasme, M. Schroeder, PLIP: fully automated protein-ligand interaction profiler. *Nucleic Acids Res.* **43**, W443-447 (2015).
23. A. Ianevski, A. K. Giri, T. Aittokallio, SynergyFinder 2.0: visual analytics of multi-drug combination synergies. *Nucleic Acids Res.* **48**, W488-W493 (2020).
24. K. Vanommeslaeghe *et al.*, CHARMM general force field: A force field for drug-like molecules compatible with the CHARMM all-atom additive biological force fields. *J. Comput. Chem.* **31**, 671-690 (2010).
25. R. Kumari, R. Kumar, C. Open Source Drug Discovery, A. Lynn, g_mmpbsa--a GROMACS tool for high-throughput MM-PBSA calculations. *J. Chem. Inf. Model.* **54**, 1951-1962 (2014).

26. B. L. Jilek *et al.*, A quantitative basis for antiretroviral therapy for HIV-1 infection. *Nat. Med.* **18**, 446-451 (2012).
27. S. E. St. John, A. D. Mesecar, Broad-spectrum non-covalent coronavirus protease inhibitors. US9975885 (2018).
28. M. Liu *et al.*, Design, synthesis, and structure-activity relationships of novel imidazo[4,5-c]pyridine derivatives as potent non-nucleoside inhibitors of hepatitis C virus NS5B. *Bioorg. Med. Chem.* **26**, 2621-2631 (2018).
29. J. Lennerstrand, C. K. Chu, R. F. Schinazi, Biochemical studies on the mechanism of human immunodeficiency virus type 1 reverse transcriptase resistance to 1-(beta-D-dioxolane)thymine triphosphate. *Antimicrob. Agents Chemother.* **51**, 2078-2084 (2007).
30. D. Tarantino *et al.*, Naphthalene-sulfonate inhibitors of human norovirus RNA-dependent RNA-polymerase. *Antiviral Res.* **102**, 23-28 (2014).
31. Y. G. Wen *et al.*, Population pharmacokinetics of blonanserin in Chinese healthy volunteers and the effect of the food intake. *Hum. Psychopharmacol. Clin. Exp.* **28**, 134-141 (2013).
32. Y. X. Zhang *et al.*, Comparative pharmacokinetics of aloe-emodin, rhein and emodin determined by liquid chromatography-mass spectrometry after oral administration of a rhubarb peony decoction and rhubarb extract to rats. *Pharmazie* **68**, 333-339 (2013).
33. P. T. Lukey *et al.*, A randomised, placebo-controlled study of omipalisib (PI3K/mTOR) in idiopathic pulmonary fibrosis. *Eur. Respir. J.* **53**, 1801992 (2019).
34. P. Munster *et al.*, First-in-human phase I study of GSK2126458, an oral pan-class I phosphatidylinositol-3-kinase inhibitor, in patients with advanced solid tumor malignancies. *Clin. Cancer Res.* **22**, 1932-1939 (2016).
35. S. C. J. Jorgensen, R. Kebriaei, L. D. Dresser, Remdesivir: review of pharmacology, pre-clinical data, and emerging clinical experience for COVID-19. *Pharmacotherapy* **40**, 659-671 (2020).
36. M. H. Kirschbaum *et al.*, A phase 1 trial dose-escalation study of tipifarnib on a week-on, week-off schedule in relapsed, refractory or high-risk myeloid leukemia. *Leukemia* **25**, 1543-1547 (2011).
37. H. U. Schulz, M. Schurer, D. Bassler, D. Weiser, Investigation of pharmacokinetic data of hypericin, pseudohypericin, hyperforin and the flavonoids quercetin and isorhamnetin revealed from single and multiple oral dose studies with a hypericum extract containing tablet in healthy male volunteers. *Arzneimittelforschung* **55**, 561-568 (2005).
38. M. T. Burger *et al.*, Identification of N-(4-((1R,3S,5S)-3-amino-5-methylcyclohexyl)pyridin-3-yl)-6-(2,6-difluorophenyl)- 5-fluoropicolinamide (PIM447), a potent and selective proviral insertion site of moloney murine leukemia (PIM) 1, 2, and 3 kinase inhibitor in clinical trials for hematological malignancies. *J. Med. Chem.* **58**, 8373-8386 (2015).
39. T. K. Bergmann *et al.*, Four phase 1 trials to evaluate the safety and pharmacokinetic profile of single and repeated dosing of SCO-101 in adult male and female volunteers. *Basic Clin. Pharmacol. Toxicol.* **127**, 329-337 (2020).
40. T. C. Chou, Derivation and properties of Michaelis-Menten type and Hill type equations for reference ligands. *J. Theor. Biol.* **59**, 253-276 (1976).
41. L. Shen, S. A. Rabi, R. F. Siliciano, A novel method for determining the inhibitory potential of anti-HIV drugs. *Trends Pharmacol. Sci.* **30**, 610-616 (2009).
42. Y. Koizumi *et al.*, Quantifying antiviral activity optimizes drug combinations against hepatitis C virus infection. *Proc. Natl. Acad. Sci. USA* **114**, 1922-1927 (2017).
